# Joint analysis of differential gene expression in multiple studies using correlation motifs

**DOI:** 10.1093/biostatistics/kxu038

**Published:** 2014-08-19

**Authors:** Yingying Wei, Toyoaki Tenzen, Hongkai Ji

**Affiliations:** Department of Biostatistics, Johns Hopkins University Bloomberg School of Public Health, Baltimore, MD, USA; Department of Statistics, The Chinese University of Hong Kong, Shatin NT, Hong Kong; Center for Regenerative Medicine, Cardiovascular Research Center, Massachusetts General Hospital, Boston, MA 02114, USA; Department of Biostatistics, Johns Hopkins University Bloomberg School of Public Health, Baltimore, MD, USA

**Keywords:** Bayes hierarchical model, Correlation motif, EM algorithm, Microarray, Multiple datasets

## Abstract

The standard methods for detecting differential gene expression are mostly designed for analyzing a single gene expression experiment. When data from multiple related gene expression studies are available, separately analyzing each study is not ideal as it may fail to detect important genes with consistent but relatively weak differential signals in multiple studies. Jointly modeling all data allows one to borrow information across studies to improve the analysis. However, a simple concordance model, in which each gene is assumed to be differential in either all studies or none of the studies, is incapable of handling genes with study-specific differential expression. In contrast, a model that naively enumerates and analyzes all possible differential patterns across studies can deal with study-specificity and allow information pooling, but the complexity of its parameter space grows exponentially as the number of studies increases. Here, we propose a *correlation motif* approach to address this dilemma. This approach searches for a small number of latent probability vectors called *correlation motifs* to capture the major correlation patterns among multiple studies. The motifs provide the basis for sharing information among studies and genes. The approach has flexibility to handle all possible study-specific differential patterns. It improves detection of differential expression and overcomes the barrier of exponential model complexity.

## 1. Introduction

Detecting differentially expressed genes is a basic task in the analysis of gene expression data. The state-of-the-art solutions to this problem, such as *limma* (Smyth, 2004), *SAM* (Tusher *and others*, 2001), edgeR (Robinson and Smyth, 2007; 2008), and DESeq (Anders and Huber, 2010), are mostly designed for analyzing data from a single experiment or study. With $1\,000\,000+$ samples stored in public databases such as Gene Expression Omnibus (GEO), it is now very common for scientists to have data from multiple related experiments or studies. An emerging problem is how one can integrate data from multiple studies to more effectively analyze differential expression.

**Fig. 1.**
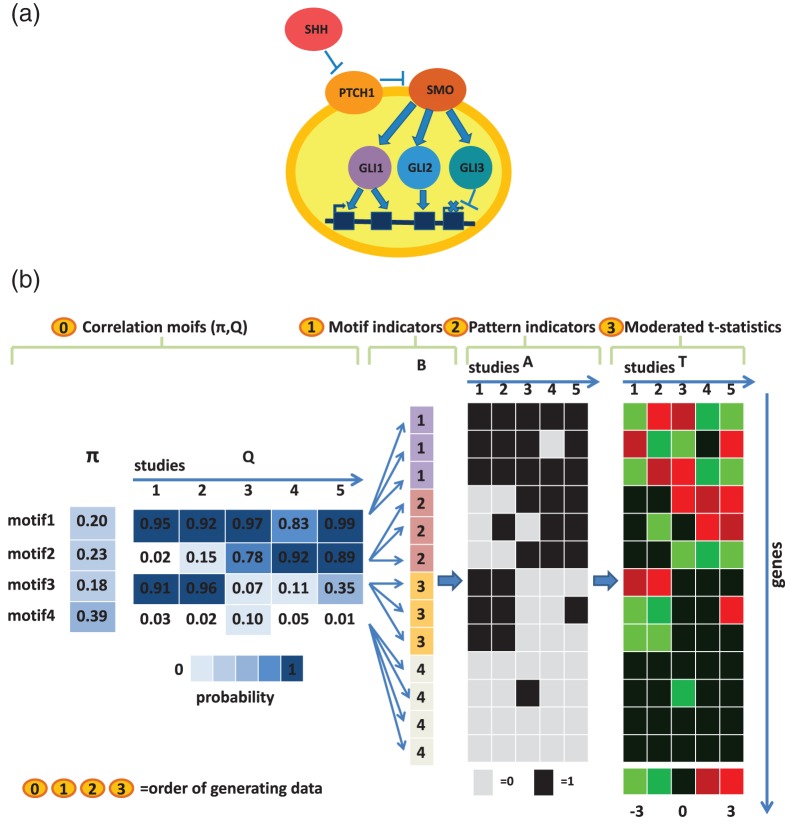
(a) A cartoon illustration of SHH pathway. (b) A numerical example of the data generating model. There exist four motifs in the dataset, with the abundance $\boldsymbol {\pi }=(0.2,0.23,0.18,0.39)$. Each row of the $\boldsymbol{Q}$ matrix represents a motif and each column corresponds to a study. Thus, $\boldsymbol{q}_{kd}$ indicates the probability for genes belonging to motif $k$ to be differentially expressed in study $d$. For example, the probability for genes belonging to motif 1 to be differentially expressed in study 4 is 0.83. The gray scale of the cells in $\boldsymbol {\pi }$ and $\boldsymbol{Q}$ illustrates the probability value. The probability increases from 0 to 1 as the color changes from light to dark. Given $\boldsymbol {\pi }$ and $\boldsymbol{Q}$, each gene is assigned a motif indicator $b_g$. For instance, the fifth gene belongs to motif 2 (indicated by a cell with a number “2”). Next, the configuration of the fifth gene, $[a_{51},a_{52},a_{53},a_{54},a_{55}]$, is generated according to $\boldsymbol{q}_2=(0.02,0.15,0.78,0.92,0.89)$. As a result, the fifth gene is differentially expressed in study 2, 4, and 5. Finally, the moderated *t*-statistic $t_{5d}$ within each study $d$ is produced according to the configuration $a_{5d}$.

One example that motivated this article is a study of the vertebrate sonic hedgehog (SHH) signaling pathway. SHH is a signaling protein that can bind to patched 1 (PTCH1), a receptor protein in cell membrane (Figure 1(a)). PTCH1 can interact with another membrane protein smoothened (SMO) to repress its activity. In the absence of SHH, PTCH1 keeps SMO inactive. The presence of SHH will repress PTCH1 and activate SMO. The active SMO triggers a signaling cascade to modulate activities of three transcription factors, GLI1, GLI2, and GLI3, which in turn induce or repress the expression of hundreds of downstream target genes. SHH pathway is a core signaling pathway in vertebrate (Ingham and McMahon, 2001). To elucidate the underlying mechanisms linking this pathway to development and diseases, multiple studies have been conducted in different contexts to identify genes whose transcriptional activities are modulated by SHH signaling. Some studies perturb the SHH signal in different tissues by knocking out or over-expressing the pathway's key signal transduction components such as SHH, PTCH1, and SMO, while others compare disease samples with corresponding controls. Table 1 contains eight such datasets in mouse originally collected by Tenzen *and others* (2006) and Mao *and others* (2006). Each dataset involves a comparison of genome-wide expression profiles between two different sample types. These data were all generated using Affymetrix Mouse Expression Set 430 arrays. The questions of biological interest include (i) which genes are controlled by the SHH signal in each dataset, (ii) which genes are the core targets that respond to the SHH signal irrespective of tissue type and developmental stage, and (iii) which genes are context-specific targets and are modulated by the SHH signal only in certain conditions.

**Table 1.** SHH microarray data description

Study ID	Condition 1 (case)	Sample No.	Condition 2 (control)	Sample No.	Reference
1	8somites_smo	3	8somites_wt	3	Tenzen *and others* (2006)
2	8somites_ptc	3	8somites_wt	3	Tenzen *and others* (2006)
3	13somites_ptc	3	13somites_wt	3	Tenzen *and others* (2006)
4	head_shh	3	head_wt	3	Tenzen *and others* (2006)
5	limb_shh	3	limb_wt	3	Tenzen *and others* (2006)
6	Medulloblastoma_tumor	3	Medulloblastoma_control	2	Mao *and others* (2006)
7	BCC_tumor	3	BCC_control	3	Mao *and others* (2006)
8	13somites_smo	3	13somites_wt	3	Tenzen *and others* (2006)

$8{\mathrm {somites}}$ and $13{\mathrm {somites}}$ indicate two different developmental stages of embryos; smo indicates mice with mutant Smo; ptc stands for mice with mutant ${\mathrm {Ptch1}};$ wt means wild type; shh represents Shh mutant. Medulloblastoma and BCC are two types of tumors.

For simplicity, below each dataset is called a *study*. One simple approach to analyze these data is to analyze each study separately using existing state-of-the-art methods such as *limma* (Smyth, 2004) or *SAM* (Tusher *and others*, 2001). This approach is not ideal as it may fail to detect genes with low-fold changes but consistently differential in many or all studies.

Modeling all data jointly may allow one to borrow information across studies to improve the analysis. A simple model to combine data is to assume that each gene is either differential in all studies or non-differential in all studies (Conlon *and others*, 2006). This concordance model may help with identifying genes with small but consistent expression changes in all studies. However, it ignores the reality that activities of many important genes are tissue- or time-specific. This method will only produce a single gene list that reports and ranks genes in the same way for all studies. It cannot prioritize genes differently for different studies to account for context-specificity.

A more flexible approach is to consider all possible differential expression patterns. Suppose there are $D$ studies and each gene can either be differential or non-differential in each study, there will be $2^D$ possible differential expression patterns. One can model the data as a mixture of $2^D$ different gene classes. This allows one to deal with context-specificity. However, an obvious drawback is that as the number of studies increases, the number of possible patterns increases exponentially. Thus, the model does not scale well with the increasing $D$.

In this article, we propose a new method, *CorMotif*, for jointly analyzing multiple studies to improve differential expression detection. This method is both flexible for handling context-specificity and scalable to increasing study number. The key idea is to use a small number of latent probability vectors called “correlation motifs” to model the major correlation patterns among the studies. The motifs essentially group genes into clusters based on their differential expression patterns, and the differential gene detection is coupled with the clustering.

Unlike *CorMotif*, many methods developed previously for analyzing differential expression in multiple studies or conditions, such as the Empirical Bayes approach by Kendziorski *and others* (2003) (called “eb1” hereinafter), the method by Jensen *and others* (2009) and the method by Ruan and Yuan (2011), have exponential model complexity and therefore limited scalability. The XDE approach proposed by Scharpf *and others* (2009) does not have explosive complexity, but it is not flexible enough to model the heterogeneity among genes in terms of their cross-study correlation patterns. These methods are reviewed in more detail in supplementary material A.1 available at *Biostatistics* online. Yuan and Kendziorski (2006) explored the idea of coupling clustering with differential expression analysis to better deal with the heterogeneity of genes. However, these authors only considered detecting differential expression between two conditions in one study. Conceptually, their approach may be combined with the model developed by Kendziorski *and others* (2003) to handle multiple studies. However, such a simple extension would lead to a model (called “eb10best” hereinafter) in which genes are assumed to fall into multiple clusters and each cluster is a mixture of $2^D$ differential patterns. Once again, the model complexity explodes as the dataset number increases. Compared with these methods, *CorMotif* offers a unique data integration solution in that it addresses study-specificity, heterogeneity among genes, and exponential complexity simultaneously. Below we focus on discussing *CorMotif* for microarray data since it was motivated by the microarray analysis in the SHH study. However, the idea behind *CorMotif* is general, and it should be straightforward to develop a similar framework for RNA-seq data.

## 2. Methods

### 2.1 Data structure and preprocessing

Suppose there are $G$ genes and $D$ microarray studies. Each study $d$ compares two biological conditions (e.g. cancer versus normal), and each condition $l$ has $n_{dl}$ replicate samples. Different studies may be related, but they can compare different biological conditions. Let $x_{gdlj}$ be the normalized and appropriately transformed expression value of gene $g$ in study $d$, condition $l,$ and replicate $j$. In this article, all data were normalized and log-transformed using RMA (Irizarry *and others*, 2003). The ensemble of observed data is $\boldsymbol{X}=\{x_{gdlj}: g=1,\ldots ,G; d=1,\ldots ,D; l=1,2; j=1,\ldots ,n_{dl}\}$.

Each gene can be differentially expressed in some, all, or none of the studies. Let $a_{gd}=1$ or $0$ indicate whether gene $g$ is differentially expressed in study $d$ or not. $\boldsymbol{A} = (a_{gd})_{G \times D}$ is a $G \times D$ matrix that contains all $a_{gd}$s. Given the observed data $\boldsymbol{X}$, one is interested in inferring $\boldsymbol{A}$.

*CorMotif* first applies limma (Smyth, 2004) to each study separately. Define $\bar {x}_{gdl}=\sum _{j} x_{gdlj}/n_{dl}$, $n_d = n_{d1}+n_{d2}$ and $v_{d}={1}/{n_{d1}}+{1}/{n_{d2}}$. For gene $g$ and study $d$, compute the mean expression difference $y_{gd}=\bar {x}_{gd1}-\bar {x}_{gd2}$ and sample variance $s_{gd}^2 = \sum _l \sum _j (x_{gdlj}-\bar {x}_{gdl})^2 / (n_d-2)$. The limma approach assumes that $y_{gd}$s and $s_{gd}^2$s within each study $d$ follow a hierarchical model: (i) $[y_{gd} | \mu _{gd}, \sigma _{gd}^2] \sim N(\mu _{gd}, v_{d} \sigma _{gd}^2)$, (ii) $\mu _{gd}=0$ if $a_{gd}=0$, (iii) $[\mu _{gd} | a_{gd}=1, \sigma _{gd}^2] \sim N(0, w_{d} \sigma _{gd}^2)$, (iv) [$s_{gd}^2 | \sigma _{gd}^2] \sim \frac {\sigma _{gd}^2}{(n_d-2)}\chi ^2_{n_d-2}$, and (v) $[{1}/{\sigma _{gd}^2}] \sim ({1}/{n_{0d}s_{0d}^2}) \chi ^2_{n_{0d}}$. Here, $w_d$, $n_{0d},$ and $s_{0d}^2$ are unknown parameters. Their values can be estimated using the procedure described in Smyth (2004). This hierarchical model allows one to pool information across genes to stabilize the variance estimates. Smyth (2004) shows that it can significantly improve differential gene detection when the sample size $n_d$ is small. For each study $d$, limma produces a moderated *t*-statistic for each gene $g$, computed as $t_{gd}=y_{gd}/\sqrt {v_{d}\tilde {s}_{gd}^2},$ where $\tilde {s}_{gd}^2 = ({n_{0d}s_{0d}^2+(n_d-2)s_{gd}^2})/({n_{0d}+n_d-2})$. This statistic summarizes gene $g$'s differential expression information in study $d$. Under this model, when gene $g$ is not differentially expressed in study $d$ (i.e. $a_{gd}=0$), $t_{gd}$ follows a *t*-distribution $t_{n_{0d}+n_d-2}$; when $a_{gd}=1$, $t_{gd}$ follows a scaled *t*-distribution $(1+w_{d}/v_d)^{1/2}t_{n_{0d}+n_d-2}$ (Smyth, 2004).

Next, we arrange all $t_{gd}$s into a matrix $\boldsymbol{T} = (t_{gd})_{G \times D}$. *CorMotif* will then use $\boldsymbol{T}$ instead of the raw expression values $\boldsymbol{X}$ to infer $\boldsymbol{A}$.

### 2.2 Correlation motif model

Organize the differential expression states of gene $g$ into a vector $\boldsymbol{a}_g=[a_{g1},a_{g2},\ldots ,a_{gD}]$. For $D$ studies, $\boldsymbol{a}_g$ has $2^D$ possible configurations. A simple way to describe the correlation among studies is to document the empirical frequency of observing each of the $2^D$ configurations of $\boldsymbol{a}_g$ among all genes. This is because $f(\boldsymbol{a}_g)$, the joint distribution of $[a_{g1},a_{g2},\ldots ,a_{gD}]$, is known once the probability of observing each configuration is given. This joint distribution will determine how $a_{gd}$s from different studies are correlated. While simple, this approach is not scalable since it requires $O(2^D)$ parameters and the parameter space expands exponentially with increasing $D$.

To avoid this limitation, *CorMotif* adopts a hierarchical mixture model (Figure 1(b)). The model assumes that genes fall into $K$ different classes ($K \ll 2^D$ for big $D$), and the moderated *t*-statistics $\boldsymbol{T} = (t_{gd})_{G\times D}$ are viewed as generated as follows. First, each gene $g$ is randomly and independently assigned a class label $b_g$ according to probability $\boldsymbol {\pi }=(\pi _1,\ldots ,\pi _K)$. Here, $\pi _k \equiv {\mathrm {Pr}}(b_g=k)$ is the prior probability that a gene belongs to class $k$, and $\sum _k \pi _k = 1$. Secondly, given genes’ class labels (i.e. $b_g$s), genes’ differential expression states $a_{gd}$s are generated independently according to probabilities $q_{kd} \equiv {\mathrm {Pr}}(a_{gd}=1|b_g=k)$. For genes in the same class $k$, $\boldsymbol{a}_g$s are generated using the same probabilities $\boldsymbol{q}_{\boldsymbol{k}} = (q_{k1},\ldots ,q_{kD})$. Thirdly, given the differential expression states $a_{gd}$s, genes’ moderated *t*-statistics $t_{gd}$s are generated independently according to $f_{d1}(t_{gd})=f(t_{gd}|a_{gd}=1) \sim (1+w_{d}/v_d)^{1/2}t_{n_{0d}+n_d-2}$ or $f_{d0}(t_{gd})=f(t_{gd}|a_{gd}=0) \sim t_{n_{0d}+n_d-2}$.

Let $\boldsymbol{B} = (b_{1},\ldots ,b_{G})$ be the class membership for all genes. Organize $\boldsymbol{q}_{\boldsymbol{k}}$ into a matrix $\boldsymbol{Q}=(\boldsymbol{q}_{\boldsymbol{1}}^{\textbf {T}},\ldots , \boldsymbol{q}_{\boldsymbol{K}}^{\textbf {T}})^{\rm T}= (q_{kd})_{K \times D}$. Let $\delta (\cdot)$ be an indicator function: $\delta (\cdot)=1$ if its argument is true, and $\delta (\cdot)=0$ otherwise. Based on the above model, the joint probability distribution of $\boldsymbol{A}$, $\boldsymbol{B}$, and $\boldsymbol{T}$ conditional on $\boldsymbol {\pi }$ and $\boldsymbol{Q}$ is

(2.1) \begin{equation*}\label{eq2.1} {\mathrm{Pr}}(\boldsymbol{T},\boldsymbol{A},\boldsymbol{B}|\boldsymbol{\pi},\boldsymbol{Q}) =\prod_{g=1}^{G}\prod_{k=1}^{K}\left\{\pi_k\prod_{d=1}^D[q_{kd}f_{d1}(t_{gd})]^{a_{gd}}[(1-q_{kd})f_{d0}(t_{gd})]^{1-a_{gd}}\right\}^{\delta{(b_g=k)}} \end{equation*}

In this model, each gene class $k$ is associated with a vector $\boldsymbol{q}_{\boldsymbol{k}}$ whose elements are the prior probabilities of a gene in this class to be differential in studies $1,\ldots ,D$. Each $\boldsymbol{q}_{\boldsymbol{k}}$ represents a probabilistic differential expression pattern and therefore is called a “motif”. Since $q_{kd}$s are probabilities, genes in the same class can have different $\boldsymbol{a}_g$ configurations. On the other hand, genes from the same class share the same $\boldsymbol{q}_{\boldsymbol{k}}$, and hence their differential expression configuration $\boldsymbol{a}_g$s tend to be similar. Genes in different classes have different $\boldsymbol{q}_{\boldsymbol{k}}$s, and their $\boldsymbol{a}_g$s also tend to be different. Essentially, our model groups genes into $K$ clusters based on $\boldsymbol{a}_g$. However, unlike an usual clustering algorithm, here $\boldsymbol{a}_g$s are unknown.

Despite the assumption that $a_{gd}$s are *a priori* independent conditional on the class label $b_g$, $a_{gd}$s are no longer independent once the class label $b_g$ is integrated out. To see this, consider the prior probability that a gene is differentially expressed in all studies. Based on our model, ${\rm Pr}(\textbf {a}_{g}=[1,\ldots ,1])=\sum _{k}(\pi _k\prod _d{q_{kd}})$, which is different from the product of the marginals $\prod _d {\mathrm {Pr}}(a_{gd}=1)=\prod _d (\sum _{k}\pi _kq_{kd})$. This explains why the hierarchical mixture model above can be used to describe the correlation among multiple studies. Since the mixture of $\boldsymbol{q}_{\boldsymbol{k}}$s provides the key to model the cross-study correlation, each vector $\boldsymbol{q}_{\boldsymbol{k}}$ is also called a “correlation motif”.

A model with $K$ correlation motifs requires $O(KD)$ parameters in total. Usually, a small $K$ ($\ll 2^D$ when $D$ is big) is sufficient to capture the major correlation structure in the real data. Therefore, our method can be easily scaled up to deal with large $D$ scenarios. When $0<q_{kd}<1$, each $\boldsymbol{q}_{\boldsymbol{k}}$ will be able to generate all $2^D$ configurations with non-zero probabilities. Thus, our model also retains the flexibility to allow all $2^D$ configurations of $\boldsymbol{a}_g$ to occur at individual gene level.

### 2.3 Statistical inference

In reality, only $\boldsymbol{T}$ is observed. $\boldsymbol {\pi }$ and $\boldsymbol{Q}$ are unknown parameters. $\boldsymbol{A}$ and $\boldsymbol{B}$ are unobserved missing data. To infer the unknowns from $\boldsymbol{T}$, we first assume that $K$ is given and introduce a Dirichlet prior ${\mathrm {Dir}}(2,\ldots ,2)$ for $\boldsymbol {\pi }$ and a Beta prior $B(2,2)$ for $q_{kd}$ (for a discussion on the choice of prior, see supplementary material A.2 available at *Biostatistics* online). As a result,

(2.2) \begin{align} {\mathrm{Pr}}(\boldsymbol{\pi},\boldsymbol{Q},\boldsymbol{A},\boldsymbol{B}|\boldsymbol{T})&\propto \prod_{g=1}^{G}\prod_{k=1}^{K}\left\{\pi_k\prod_{d=1}^D[q_{kd}f_{d1}(t_{gd})]^{a_{gd}}[(1-q_{kd})f_{d0}(t_{gd})]^{1-a_{gd}}\right\}^{\delta{(b_g=k)}}\nonumber\\ &\quad *\prod_{k=1}^K\pi_k\prod_{k=1}^K\prod_{d=1}^Dq_{kd}(1-q_{kd})\label{eq2.2} \end{align}

Based on the above posterior distribution, an expectation–maximization (EM) algorithm (Gelman *and others*, 2004) can be derived to search for the posterior mode of $\boldsymbol {\pi }$ and $\boldsymbol{Q}$.

Using the estimated $\hat {\boldsymbol {\pi }}$ and $\hat {\boldsymbol{Q}}$, one can then compute $E(a_{gd} | \boldsymbol{T}, \hat {\boldsymbol {\pi }}, \hat {\boldsymbol{Q}}) = {\mathrm {Pr}}(a_{gd} = 1| \boldsymbol{T}, \hat {\boldsymbol {\pi }}, \hat {\boldsymbol{Q}})$, the posterior probability that gene $g$ is differentially expressed in study $d$ after integrating out the motif membership $b_g$. Next, we rank-order genes in each study separately using ${\rm Pr}(a_{gd} = 1| \boldsymbol{T}, \hat {\boldsymbol {\pi }}, \hat {\boldsymbol{Q}})$. The ranked lists can be used to choose follow-up targets. Users can also provide a posterior probability cutoff to dichotomize genes into *differential* or *non-differential* genes in each study. The default cutoff is 0.5. Users have the option to set the cutoff to other values.

In order to choose the motif number $K$, we use Bayesian Information Criterion (BIC). Details of the EM algorithm and how to use BIC to choose $K$ are provided in the supplementary material A.3 and A.4 available at *Biostatistics* online.

*CorMotif* improves the differential expression detection by integrating information both across studies and across genes. ${\mathrm {Pr}}(a_{gd} = 1| \boldsymbol{T}, \hat {\boldsymbol {\pi }}, \hat {\boldsymbol{Q}})$ can be decomposed as $\sum _{k=1}^K {\mathrm {Pr}}(a_{gd} = 1| \boldsymbol{T}, \hat {\boldsymbol {\pi }}, \hat {\boldsymbol{Q}}, b_g=k)*{\rm Pr}(b_g = k| \boldsymbol{T}, \hat {\boldsymbol {\pi }}, \hat {\boldsymbol{Q}})$. Here, ${\mathrm {Pr}}(b_g = k| \boldsymbol{T}, \hat {\boldsymbol {\pi }}, \hat {\boldsymbol{Q}})$ is determined by jointly evaluating gene $g$'s data in all studies, and ${\mathrm {Pr}}(a_{gd} = 1| \boldsymbol{T}, \hat {\boldsymbol {\pi }}, \hat {\boldsymbol{Q}}, b_g=k)$ contains information specific to study $d$. According to Bayes’ theorem, ${\mathrm {Pr}}(a_{gd} = 1 | \boldsymbol{T}, \hat {\boldsymbol {\pi }}, \hat {\boldsymbol{Q}}, b_g=k) \propto {\rm Pr}(t_{gd} | a_{gd} = 1, \hat {\boldsymbol {\pi }}, \hat {\boldsymbol{Q}}, b_g=k) \times {\rm Pr}(a_{gd} = 1 | \hat {\boldsymbol {\pi }}, \hat {\boldsymbol{Q}}, b_g=k)$. $t_{gd}$ in the first term contains expression information for a given gene $g$ in study $d$. To compute its denominator, the limma approach also utilized information across genes to help with estimating the variance. Meanwhile, the second term ${\mathrm {Pr}}(a_{gd} = 1 | \hat {\boldsymbol {\pi }}, \hat {\boldsymbol{Q}}, b_g=k)$ involves prior probabilities given by the correlation motifs (i.e. $\hat {\boldsymbol{q}}_{\boldsymbol{k}}$s) which are estimated using data from all genes. Owing to this two-way information pooling (i.e. across both studies and genes), *CorMotif* uses information more effectively than methods based on only a single gene or a single study. This is especially useful for analyzing studies with relatively weak signal-to-noise ratio.

## 3. Simulations

### 3.1 Compared methods

We compared *CorMotif* with six other methods: *separate limma*, *all concord*, *full motif*, *SAM*, *eb1*, and *eb10best*. We did not compare the method in Jensen *and others* (2009) as no software was available for this method. The *separate limma* approach analyzes each study separately using limma. The moderated *t*-statistics in each study are assumed to be a mixture of $t_{n_{0d}+n_d-2}$ and $(1+w_{d}/v_d)^{1/2}t_{n_{0d}+n_d-2}$. To better evaluate the gain from data integration, we matched this analysis to *CorMotif* as much as possible by running an EM algorithm similar to *CorMotif* to compute the posterior probability for differential expression using 0.5 as default cutoff. Conceptually, this makes *separate limma* equivalent to *CorMotif* with a single cluster ($K=1$), and the analysis produces the same gene ranking as limma in each study. *All concord* assumes that a gene is either differential in all studies or non-differential in all studies (i.e. $\boldsymbol{a}_g = [1,1,\ldots ,1]$ or $[0,0,\ldots ,0]$). Conditional on $\boldsymbol{a}_g$, the model for $t_{gd}$ remains the same as *CorMotif* and limma. *Full motif* assumes that genes fall into $2^D$ classes, corresponding to the $2^D$ possible $\boldsymbol{a}_g$ configurations. It can be viewed as a saturated version of *CorMotif*. All the other methods are applied to $x_{gdlj}$s directly. *SAM* (Tusher *and others*, 2001) processes each study separately, whereas *eb1* and *eb10best* analyze all studies jointly. The *eb1* method corresponds to the R package EBarrays with lognormal–normal (LNN) and one cluster assumption (Kendziorski *and others*, 2003). The *eb10best* method is EBarrays with LNN and multiple cluster assumption, and the cluster number is chosen by EBarrays as the one with the lowest AIC (Yuan and Kendziorski, 2006). We also tried XDE (Scharpf *and others*, 2009). However, it is based on Markov Chain Monte Carlo (MCMC) and took extremely long computing time, usually 24 h on a machine with 2.7 GHz CPU and 4 Gb RAM for 1000 iterations, for an analysis involving four studies which was the smallest data we analyzed here. Moreover, 1000 iterations usually were not enough for XDE to converge. Therefore, XDE will not be compared hereinafter. *eb10best* failed to work when it was used to jointly analyze $\geq 7$ studies. *Full motif* and *eb1* failed when there were 20 studies.

### 3.2 Model-based simulations

**Fig. 2.**
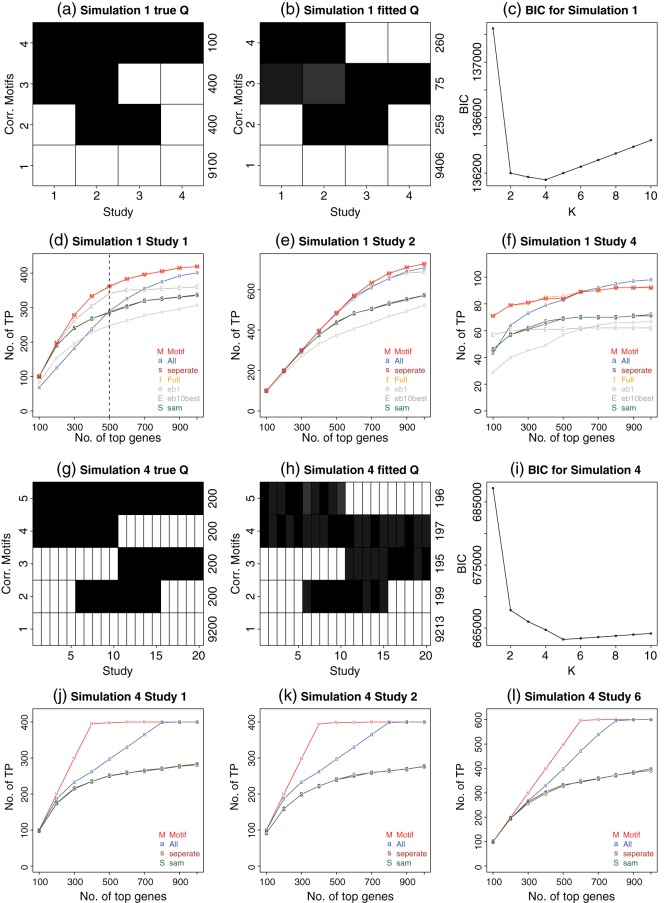
Results for the model assumption-based simulations (simulations 1 and 4). Also see supplemental Figure A.1 available at *Biostatistics* online for simulations 2 and 3. (a) and (g) True motif patterns for simulations 1 and 4. The $\boldsymbol{Q}$ of the true motifs is shown. Each row indicates a motif pattern and each column represents a study. The actual number of genes belonging to each motif (i.e. $\boldsymbol {\pi }*G$) is displayed at the right end of each row. The gray scale of the cell $(k,d)$ demonstrates the probability of differential expression in study $d$ for pattern $k$. Black means 1 and white means 0. (b) and (h) The estimated $\hat {\boldsymbol{Q}}$ from the learned motifs with $\hat {\boldsymbol {\pi }}*G$ annotated at the end of each row. (c) and (i) BIC plots. It can be seen that motif patterns reported by *CorMotif* under the minimal BIC are similar to the true underlying motif patterns. (d)–(f) and (j)–(l) Gene ranking performance of different methods in simulations 1 and 4. ${\mathrm {TP}}_d(r)$, the number of genes that are truly differentially expressed in study $d$ among the top $r$ ranked genes by a given method, is plotted against the rank cutoff $r$. For each simulation, results for a few representative studies are shown. Each plot is for one study.

We first tested *CorMotif* using simulations. In simulation 1, we generated 10 000 genes and four studies according to the four differential patterns in Figure 2(a): 100 genes were differentially expressed in all four studies ($\boldsymbol{a}_g = [1,1,1,1]$); 400 genes were differential only in studies 1 and 2 ($[1,1,0,0]$); 400 genes were differential only in studies 2 and 3 ($[0,1,1,0]$); 9100 genes were non-differential ($[0,0,0,0]$). Each study had six samples: three cases and three controls. The variances $\sigma _{gd}^2$s were simulated from a scaled inverse $\chi ^2$ distribution $n_{0d}s_{0d}^2/\chi ^2(n_{0d})$, where $n_{0d}=4$ and $s_{0d}^2=0.02$. Given $\sigma _{gd}^2$, the expression values were generated using $x_{gdlj} \sim N(0,\sigma _{gd}^2$). Whenever $a_{gd}=1$, we drew $\mu _{gd}$ from $N(0,w_{0d}*\sigma _{gd}^2),$ where $w_{0d}=4$, and $\mu _{gd}$ was then added to the expression values of the three cases (i.e. $x_{gd1j}$s).

*CorMotif* was fit with varying motif number $K$. As Figure 2(c) shows, the minimal BIC was achieved at $K=4$. As a result, four motifs were reported (Figure 2(b)). The reported motifs were very similar to the true underlying differential patterns in Figure 2(a).

Different methods were then compared in terms of how good they rank differential genes in each individual study (Figure 2(d)–(f)) as well as how accurate they can infer each gene's differential configuration $\boldsymbol{a}_g$ in all studies (Table 2). For each study $d$, *CorMotif* ranks genes using the posterior probability ${\mathrm {Pr}}(a_{gd} = 1| \boldsymbol{T}, \hat {\boldsymbol {\pi }}, \hat {\boldsymbol{Q}})$ which is obtained after integrating out the motif membership $b_g$. A gene was called differential in study $d$ (i.e. $\hat {a}_{gd}=1$) if ${\mathrm {Pr}}(a_{gd} = 1| \boldsymbol{T}, \hat {\boldsymbol {\pi }}, \hat {\boldsymbol{Q}})>0.5$. Both the gene rankings and differential expression calls were different for different studies since ${\mathrm {Pr}}(a_{gd} = 1| \boldsymbol{T}, \hat {\boldsymbol {\pi }}, \hat {\boldsymbol{Q}})$ depends on $d$ and can change across studies. This is a desirable property as in reality the sets of true differential genes may be different in different studies due to study-specific differential expression, and ultimately one wants to know which genes are differential in each study. Using a similar approach, we obtained gene rankings and differential calls for *full motif*, *eb1* and *eb10best* which were also study-specific. *Separate limma* and *SAM* analyze each study separately and naturally produce study-specific gene ranking and differential calls. For all the methods above, we did not combine differential calls of a gene in $D$ studies into a single call to indicate whether the gene is differential in any study, nor did we use such a combined call to rank genes, since the combined call would fail to capture study-specificity. Unlike the other methods, *all concord* assumes common differential states in all studies, therefore its gene ranking and differential calls remain the same across studies.

**Table 2.** Confusion matrix for simulation $1$

Method	Differential configuration	$c(0,0,0,0)$	$c(0,1,1,0)$	$c(1,1,0,0)$	$c(1,1,1,1)$
*CorMotif*	$c(0,0,0,0)$	9072	161	165	16
	$c(0,1,1,0)$	3	168	3	7
	$c(1,1,0,0)$	3	2	151	6
	$c(1,1,1,1)$	0	1	0	33
	${\mathrm {other}}$	22	68	81	38
*separate limma*	$c(0,0,0,0)$	9035	144	144	16
	$c(0,1,1,0)$	0	68	0	5
	$c(1,1,0,0)$	0	0	57	6
	$c(1,1,1,1)$	0	0	0	4
	${\mathrm {other}}$	65	188	199	69
*all concord*	$c(0,0,0,0)$	9095	236	236	20
	$c(0,1,1,0)$	0	0	0	0
	$c(1,1,0,0)$	0	0	0	0
	$c(1,1,1,1)$	5	164	164	80
	${\mathrm {other}}$	0	0	0	0
*full motif*	$c(0,0,0,0)$	9072	161	164	16
	$c(0,1,1,0)$	4	172	4	7
	$c(1,1,0,0)$	3	2	155	6
	$c(1,1,1,1)$	0	1	0	35
	${\mathrm {other}}$	21	64	77	36
*eb1*	$c(0,0,0,0)$	62	0	2	0
	$c(0,1,1,0)$	2178	30	22	3
	$c(1,1,0,0)$	569	7	12	0
	$c(1,1,1,1)$	753	34	32	64
	${\mathrm {other}}$	5538	329	332	33
*eb10best*	$c(0,0,0,0)$	0	0	0	1
	$c(0,1,1,0)$	316	220	16	10
	$c(1,1,0,0)$	180	23	226	10
	$c(1,1,1,1)$	5789	77	52	63
	${\mathrm {other}}$	2815	80	106	16
*SAM*	$c(0,0,0,0)$	9099	256	279	48
	$c(0,1,1,0)$	0	20	0	3
	$c(1,1,0,0)$	0	0	9	2
	$c(1,1,1,1)$	0	0	0	1
	${\mathrm {other}}$	1	124	112	46

The column labels indicate the true underlying patterns and the row labels represent the reported configurations at gene level. For *CorMotif*, *separate limma*, *all concord*, *full motif*, *eb1*, and *eb10best*, differential expression in each study is determined using their default posterior probability cutoff $0.5$. For *SAM*, *q*-value cutoff $0.1$ was used to call differential expression. This yields similar number of correct classifications for pattern $[0,0,0,0]$ compared with *CorMotif*.

To examine if *CorMotif* can improve gene ranking, in each study and for each method we counted the number of true differential genes (true positives), ${\mathrm {TP}}_d(r)$, among the top $r$ ranked genes, and we plotted ${\rm TP}_d(r)$ versus $r$ in Figure 2(d)–(f). *CorMotif* consistently performed among the best in all studies. For instance, Figure 2(d) shows the results for study 1. *CorMotif* identified 361 true differential genes among its top 500 gene list. This performance was almost the same as the saturated model *full motif* which identified 362 true positives among the top 500 genes. Among the other methods, *eb10best* identified 341, *all concord* identified 292, and the others identified fewer than 292 true positives among the top 500 genes. Thus, *CorMotif* detected at least 23.6% more true positives compared with any other method except *full motif* and *eb10best*. Similarly, among the top 1000 genes, *CorMotif* and *full motif* both identified 419 true positives, *all concord* identified 401, *eb10best* identified 360, and the other methods identified fewer than 337. *CorMotif* and *full motif* detected 4.5% more true positives compared with *all concord* and improved the ranking by at least 16.4% compared with *eb10best* and other methods. Both *full motif* and *eb10best* have the problem of exponentially growing parameter space. As we will show later, they both will break down when the study number $D$ is large.

To test whether *CorMotif* can more accurately determine a gene's differential configuration, we constructed the confusion matrix in Table 2. For each gene, its binary differential calls $a_{gd}$s based on ${\mathrm {Pr}}(a_{gd} = 1| \boldsymbol{T}, \hat {\boldsymbol {\pi }}, \hat {\boldsymbol{Q}})$ in different studies were arranged into a vector to represent its estimated differential configuration $\boldsymbol{a}_g$. For *CorMotif*, *separate limma*, *all concord*, *full motif*, *eb1* and *eb10best*, differential expression was called using their default posterior probability cutoff 0.5. For *SAM*, *q*-value cutoff 0.1 was used to call differential expression. At this cutoff, *SAM* correctly identified similar number of genes with $\boldsymbol{a}_g = [0,0,0,0]$ (i.e. non-differential in all studies) compared with *CorMotif*. This allowed us to meaningfully compare *SAM* and *CorMotif* in terms of their ability to find differential genes. Table 2 shows that *CorMotif* was better at characterizing genes’ true differential configurations compared with most other methods. For instance, among the 400 $[0,1,1,0]$, 400 $[1,1,0,0],$ and 100 $[1,1,1,1]$ genes, *CorMotif* correctly reported differential label $a_{gd}$ in all four studies for 168, 151, and 33 genes, respectively. In contrast, *separate limma* only unmistakenly labeled 68, 57, and 4 genes, respectively. Here, the increased power by *CorMotif* was purely due to the use of correlation motifs to integrate multiple studies, since all other model assumptions made by *CorMotif* and *separate limma* are the same. *All concord* requires genes to have the same differential status in all studies. As such, it is powerful at identifying concordant signals across studies but lacks the flexibility to handle study-specific differential expression: it correctly identified 80 out of 100 $[1,1,1,1]$ genes, but none of the $[0,1,1,0]$ and $[1,1,0,0]$ genes were correctly labeled as study-specific. With the default cutoff, *eb1* and *eb10best* only labeled 62 and 0 out of 9100 $[0,0,0,0]$ genes as completely non-differential, compared with 9072 labeled by *CorMotif*. In other words, *eb1* and *eb10best* reported more false-positive differential events. Both were anti-conservative. At the same time, fewer $[0,1,1,0]$ and $[1,1,0,0]$ genes were correctly identified by *eb1* (30 and 12 versus 168 and 151 by *CorMotif*). *SAM* was also poor at identifying the differential patterns $[1,1,1,1]$, $[1,1,0,0]$, and $[0,1,1,0]$ but behaved more conservatively by labeling many of them as $[0,0,0,0]$. Among all the methods, only *full motif* performed slightly better than *CorMotif*. Even so, *CorMotif* was able to perform close to this saturated model. Adding up the diagonal elements in the confusion matrix, *CorMotif* unmistakenly assigned $\boldsymbol{a}_g$ labels to 9424 genes, whereas this number was 9164 for *separate limma*, 9175 for *all concord*, 9434 for *full motif*, 168 for *eb1*, 509 for *eb10best*, and 9129 for *SAM*.

Using a similar approach, we performed simulations 2–4 which involved different study numbers and differential expression patterns. The complete results are shown in Figure 2, see supplementary material Figure A.1 and Tables A.1–A.3 available at *Biostatistics* online. The conclusions were similar to simulation 1. In many cases, the gain brought by *CorMotif* was substantial (e.g. Figure 2(j)–(l), see supplementary material Figure A.1(j) and (k) available at *Biostatistics* online). In particular, simulation 4 had 20 studies. *full motif*, *eb1* and *eb10best* all failed to run on this data, whereas *CorMotif* was still able to borrow information across studies (Figure 2(g)–(l)).

### 3.3 Simulations based on real data

In real data, the distributions for $x_{gdlj}$s may deviate from our model assumptions. Therefore, we further evaluated *CorMotif* using simulations that retained the real data noise structure. In simulation 5, 24 Human U133 Plus 2.0 Affymetrix microarray samples were downloaded from four GEO experiments. Each experiment corresponds to a different tissue and consists of six biological replicates (see supplementary material Table A.4 available at *Biostatistics* online). After RMA normalization, replicate samples in each experiment were split into three “cases” and three “controls”. We then spiked in differential signals by adding random $N(0,1)$ deviates to the three cases according to patterns shown in supplementary material Figure A.2(a) available at *Biostatistics* online. Data simulated in this way were able to keep the background characteristics in real data. Simulation 5 is similar to simulations 1 and 2. *CorMotif* again recovered the underlying differential patterns (see supplementary material Figure A.2(b) and (c) available at *Biostatistics* online). It showed comparable differential gene detection performance to *full motif* and outperformed the other methods (see supplementary material Figure A.3(a)–(c) and Table A.5 available at *Biostatistics* online). In a similar fashion, we performed simulations 6 and 7 based on real data (see supplementary material A.5 available at *Biostatistics* online). These two simulations have the same differential signal patterns as simulations 3 and 4, respectively. Here, the motifs reported by *CorMotif* differ slightly from the underlying truth, but all the major correlation patterns were captured by the reported motifs (see supplementary material Figure A.2 available at *Biostatistics* online). Once again, *CorMotif* performed the best in terms of differential gene detection (see supplementary material Figure A.3 and Tables A.6–A.7 available at *Biostatistics* online), and *eb1*, *eb10best* and *full motif* failed to run when the study number increased (when they failed, their results were not shown).

### 3.4 Motifs are parsimonious representation of true correlation structures

As we use probability vectors to serve as motifs, it is possible that multiple weak patterns can be merged into a single motif. For instance, two complementary patterns [1,1,0,0] and [0,0,1,1] each with $n$ genes can be absorbed into a single motif with $\boldsymbol{q}_{\boldsymbol{k}}=(0.5,0.5,0.5,0.5)$ having $2n$ genes. To illustrate, we conducted simulations 8–10 which were composed of the same samples as in simulation 5 and various proportions of differential expression patterns (see supplementary material Figure A.4 available at *Biostatistics* online). In simulation 9 (see supplementary material Figure A.4(i)–(l) available at *Biostatistics* online), the relative abundance of two complementary block motifs ([1,1,0,0] and [0,0,1,1]) was small compared with the concordance motif [1,1,1,1], and they were absorbed into a single motif. In simulations 5, 8, and 10 (see supplementary material Figure A.4(a)–(h) and (m)–(p) available at *Biostatistics* online), the complementary block motifs were more abundant, and the program successfully identified them as separate motifs. In general, we observed that weaker patterns were more likely to be merged than patterns with abundant data support. In all cases, however, *CorMotif* still provided the best gene ranking results compared with other methods (see supplementary material Figure A.5 available at *Biostatistics* online). Supplementary material Figures A.4 and A.5 available at *Biostatistics* online also show that the higher the proportions of study-specific motifs (e.g. [1,1,0,0] and [0,0,1,1]), the better *CorMotif* will perform compared with the concordance analysis (i.e. *all concord*) in terms of ranking genes in each study. Together, the analyses here demonstrate that the correlation motifs only represent a parsimonious representation of the correlation structure supported by the available data. One should not expect *CorMotif* to always recover all the true underlying clusters exactly. In spite of this, our simulations show that *CorMotif* can still effectively utilize the correlation among studies to improve differential gene detection.

## 4. Application to the Shh signaling data sets

We used *CorMotif* to analyze the SHH data in Table 1. The normalized data are available for download as supplementary material Table A.10 available at *Biostatistics* online. Datasets 1 and 2 compare SMO mutant mice with wild type mice (wt) and PTCH1 mutant with wild type, respectively, in the 8 somite stage of developing embryos. Dataset 3 compares PTCH1 mutant with wild type in 13 somite stage. Datasets 4 and 5 compare SHH mutant with wild type in developing head and limb, respectively. Datasets 6 and 7 study gene expression changes in two SHH-related tumors, medulloblastoma and basal cell carcinoma (BCC), compared with normal samples (control). Dataset 8 compares SMO mutant with wild type in the 13 somite stage of developing embryos. *CorMotif* was applied to datasets 1–7. Dataset 8 was reserved for testing.

**Fig. 3.**
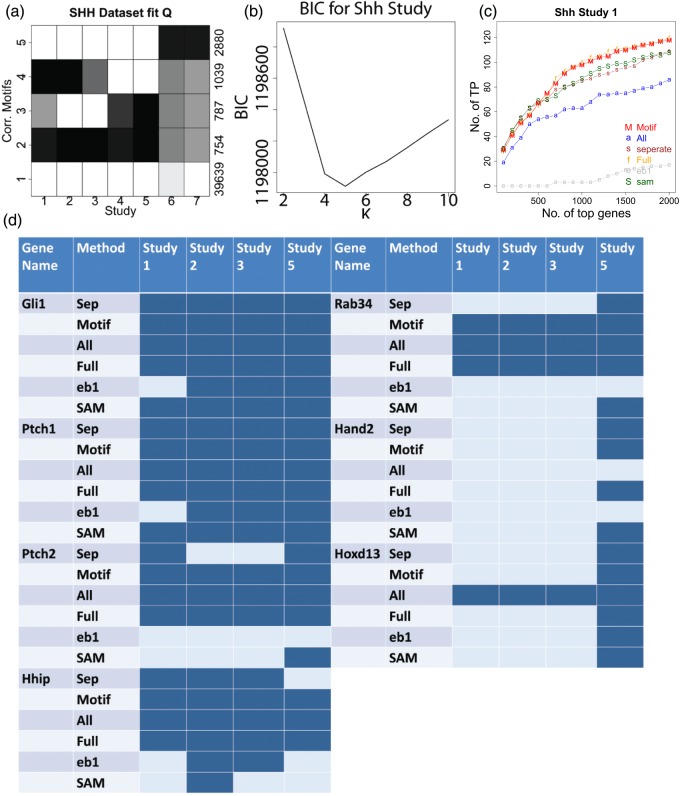
Results for the SHH data. (a) Motif patterns learned from the SHH data composed of 7 studies. (b) BIC plots for the SHH data. (c) Gene ranking performance for SHH study 1. The genes differentially expressed in dataset 8 (13somites_smo versus 13somites_wt) were obtained using *separate limma*. They were used as the gold standard. ${\mathrm {TP}}_d(r)$, the number of genes in dataset 1 that are truly differentially expressed among the top $r$ ranked genes by each method, is plotted against the rank cutoff $r$. (d) Differential status claimed by each method for known SHH pathway genes. Dark color indicates differential expression and light color represents non-differential expression.

Five motifs were discovered (Figure 3(a) and (b)). Motif 1 mainly represents background. Motif 2 contains genes that have high probability to be differential in all studies. Genes in motif 3 tend to be differential in most studies except for the two involving PTCH1 mutant (i.e. studies 2 and 3). Most genes in motif 4 are not differential in the two studies involving the SHH mutant (i.e. studies 4 and 5) but tend to be differential in all other studies. Motif 5 mainly represents genes differential in tumors (i.e. studies 6 and 7) but not in embryonic development (i.e. studies 1–5). In general, looking at the columns in Figure 3(a), the two studies involving tumors (6,7) are more similar to each other compared with other studies. The two PTCH1 mutant studies (2,3) are also relatively similar, and the same trend holds true for the two SHH mutant studies (4,5).

In this real data analysis, no comprehensive truth is available for evaluating differential expression calls. Without comprehensive knowledge about the true differential expression states of all genes in all cell types, we can only perform a partial evaluation based on existing knowledge. In this regard, we used dataset 8 as a test. Similar to dataset 1, this dataset compares SMO mutant with wild type. One expects that differential genes in these two datasets should be largely similar. Therefore, we used the top 217 differentially expressed genes detected by *separate limma* (at the posterior probability cutoff 0.5) in dataset 8 as gold standard to evaluate the gene ranking performance of different methods in dataset 1. Figure 3(c) shows that *CorMotif* again performed similar to *full motif* and outperformed all other methods. *eb10best* failed to run here. We note that since dataset 8 and datasets 2–7 represent more different biological contexts, one cannot use it as gold standard for evaluating these other datasets.

Finally, we examined well-studied SHH responsive target genes. Gli1, Ptch1, Ptch2, Hhip, and Rab34 are known to be regulated by SHH in somites and developing limb (Vokes *and others*, 2007, 2008). Therefore, we expect them to be differential in studies 1, 2, 3, and 5. Figure 3(d) shows that *CorMotif*, *all concord* and *full motif* were able to correctly identify differential expression of these genes in all these studies, whereas *separate limma*, *SAM*, and *eb1* failed to do so (they missed some cases). Supplementary material Table A.8 available at *Biostatistics* online also shows that in many studies, *CorMotif*, *all concord*, and *full motif* provided better rank for these genes compared with *separate limma*, *SAM*, and *eb1*. Hand2 is known to be a SHH target in developing limb but not in somites (Vokes *and others*, 2008). While *separate limma*, *CorMotif*, *full motif*, and *SAM* can correctly identify this, *all concord* and *eb1* failed to do so. For *all concord*, since Hand2 was not differential in studies 1–4, 6, and 7, the method thinks that this gene is not differential in any study. Similarly, Hoxd13 is a limb specific target of SHH signaling (Vokes *and others*, 2008). While the other methods correctly identified this, *all concord* failed again by claiming it to be differential in all studies. In all the genes examined, only *CorMotif* and *full motif* were able to correctly identify all known differential states.

## 5. Discussion

Together, our analyses show that *CorMotif* offers unique advantage over the other methods in the integrative analysis of multiple gene expression studies. Besides its ability to increase statistical power by combining information across studies, *CorMotif* is also flexible and scalable. Using a few probability vectors instead of $2^D$ dichotomous vectors to characterize the differential expression patterns provides the key to avoid the exponential growth of parameter space as the study number increases. At the same time, the probabilistic nature of the motifs allows all $2^D$ differential patterns to occur in the data at individual gene level.

The motif matrix ***Q*** can be viewed in two different ways. Each row of ***Q*** represents a cluster of genes with similar differential expression patterns across studies. Having many different motifs in $\boldsymbol{Q}$ is an indication that a concordance model, such as *all concord*, may not be enough to describe the correlation structure in the data. On the other hand, each column of $\boldsymbol{Q}$ represents differential expression propensities of different gene classes in a given study. If two columns are similar, the corresponding studies share similar differential expression profiles (e.g. studies 6 and 7 in the SHH data are more similar to each other compared with the other studies).

Currently, *CorMotif* first computes moderated *t*-statistics $\boldsymbol{T}$ and then applies the correlation motif model to $\boldsymbol{T}$. We used this two-stage approach for considerations of effective presentation, computational efficiency, and clean method comparison (see supplementary material A.6 available at *Biostatistics* online for a detailed discussion). The present two-stage framework is also very general. For instance, conceptually one can modify $f_{d0}$ and $f_{d1}$ to accommodate other data types such as RNA-seq. A systematic treatment of RNA-seq analysis, though, is beyond the scope of this paper. The EM implementation of *CorMotif* is computationally tractable. On a single CPU, it took $\sim $0.35 h to analyze the SHH data for a single $K$, and $2.43$ h in total in order to search for the optimal $K$ (see supplementary material A.7 and Table A.9 available at *Biostatistics* online for comparisons with other methods).

In the future, *CorMotif* may be extended in multiple ways. For example, instead of using moderated *t*-statistics and the two-stage design, one may develop a single coherent model that couples correlation motifs with a more sophisticated model for the raw data $\boldsymbol{X}$. Also, it remains to be investigated whether the problem of choosing motif number can be better dealt with by a fully Bayesian approach such as by imposing a Dirichlet Process prior for $K$ or using a variant of Dirichlet Process prior instead of using BIC. A fully Bayesian model, however, may require MCMC in the implementation, and this may pose additional challenges for developing computationally efficient algorithms capable of handling large datasets.

## 6. Software

*CorMotif* is freely available as an R package in Bioconductor: http://www.bioconductor.org/packages/release/bioc/html/Cormotif.html.

## Supplementary material

Supplementary material is available at http://biostatistics.oxfordjournals.org.

## Funding

The research is supported by the National Institutes of Health grant R01HG006282. Funding to pay the Open Access publication charges for this article was provided by the National Institutes of Health grant R01HG006282.

## Supplementary Material

Supplementary Data
