# Supplementary material for: Joint analysis of differential gene expression in multiple studies using correlation motifs
Source: Biostatistics. 2014 Aug 19;16(1):31–46. doi: 10.1093/biostatistics/kxu038 (PMC4263229; doi:10.1093/biostatistics/kxu038)
Supplement: Supplementary Data [file supp_16_1_31__index.html]

Joint analysis of differential gene expression in multiple studies using correlation motifs — Supplementary Data 

# Joint analysis of differential gene expression in multiple studies using correlation motifs

## Supplementary Data

Supplementary Data

**Files in this Supplementary Material:**

- Supplementary Data - Pdf file
- Supplementary TableA10 - csv file
