# Supplementary material for: Joint analysis of differential gene expression in multiple studies using correlation motifs
Source: Biostatistics. 2014 Aug 19;16(1):31–46. doi: 10.1093/biostatistics/kxu038 (PMC4263229; doi:10.1093/biostatistics/kxu038)
Supplement: Supplementary Data [file supp_kxu038_kxu038supp.pdf]

Biostatistics (2014), **0**, 0, *pp.* A.1–A.24
doi:10.1093/biostatistics/supp˙manuscript˙REV

Supplementary Materials for Joint Analysis of Differential Gene Expression in Multiple Studies using Correlation Motifs

YINGYING WEI
Department of Biostatistics, Johns Hopkins University Bloomberg School of Public Health, Baltimore, Maryland, USA

TOYOAKI TENZEN
Center for Regenerative Medicine, Cardiovascular Research Center, Massachusetts General Hospital, Boston, MA 02114, USA

HONGKAI JI*
Department of Biostatistics, Johns Hopkins University Bloomberg School of Public Health, Baltimore, Maryland, USA

hji@jhsph.edu

A.1. A detailed discussion on existing methods for differential expression detection in multiple studies

Previously, Kendziorski *and others* (2003) proposed an Empirical Bayes approach (called "eb1" in this article) for analyzing differential expression involving multiple biological conditions. This approach requires users to specify all possible differential patterns, and the data are then modeled accordingly. If a user applies this method to detect differential expression between two conditions in multiple studies and wants to accommodate all possible differential patterns, the user has to enumerate all 2^D possible patterns, leading to the exponential complexity problem. Similar to Kendziorski *and others* (2003), Jensen *and others* (2009) developed a hierarchical Bayesian

*To whom correspondence should be addressed.

© The Author 2014. Published by Oxford University Press. All rights reserved. For permissions, please e-mail: journals.permissions@oup.com

model and a Markov Chain Monte Carlo (MCMC) algorithm to analyze multiple conditions, again with exponential complexity due to requirement of enumerating all possible patterns. Ruan and Yuan (2011) generalizes Kendziorski *and others* (2003) to a model that can integrate information from multiple studies where each study may involve comparisons of multiple conditions. Within each study, this method enumerates all possible combinatorial patterns among multiple conditions, again resulting in exponential complexity. Moreover, differential expression patterns are assumed to be concordant across studies, that is, each gene is assumed to have the same differential pattern in all studies. The concordance assumption does not allow study-specific differential expression.

Scharpf *and others* (2009) proposed a fully Bayesian framework, XDE, for cross-study differential expression analysis. It offers two implementations. The "Single-Indicator" implementation uses a concordance model by assuming that each gene's differential state is the same across all studies. The "Multiple-Indicator" implementation allows study-specific differential expression. However, it assumes that all genes have the same prior probability to be differential within the same study, and the differential states of each gene in different studies are a priori independent. Conceptually, these assumptions are similar to a *CorMotif* model with a single cluster, which often is insufficient to capture the heterogeneity among genes since the cross-study correlation pattern may vary from one gene to another. XDE does not have the exponential complexity problem, but it uses MCMC for posterior inference and is very slow computationally.

To capture the heterogeneity among genes, Yuan and Kendziorski (2006) developed a method for simultaneous clustering and differential expression analysis. Similar to *CorMotif*, this method also assumes that genes belong to multiple clusters, and different clusters have different propensities to show differential expression. However, Yuan and Kendziorski (2006) only considered detecting differential expression between two conditions in one study. Although one may conceptually extend this approach to handle multiple studies by combining it with the model developed

by Kendziorski *and others* (2003), such a simple extension would lead to the model “eb10best” in which genes are assumed to fall into multiple clusters and each cluster is a mixture of 2^D differential patterns. As a result, the complexity of the parameter space would become $O(K*2^D)$ where K is the number of clusters.

Compared to these methods, *CorMotif* offers a unique data integration solution in that it addresses study-specificity, heterogeneity among genes, and exponential complexity simultaneously.

A.2. The choice of prior distributions

We chose the Dirichlet distribution $Dir(2, ..., 2)$ instead of $Dir(1, ..., 1)$ as the prior for $\boldsymbol{\pi}$ since the mode of a Dirichlet distribution $Dir(\alpha_1, ..., \alpha_K)$ for the mth component is $(\alpha_m - 1)/(\sum_{k=1}^{K} \alpha_k - K)$, which is zero when $\alpha_m = 1$ and not defined when all α_ks are equal to one. As a result, in the EM iterations, when a motif is associated with very few genes such that $\sum_{g=1}^{G} E(\delta(b_g = m)|\boldsymbol{T}, \hat{\boldsymbol{\pi}}, \hat{\boldsymbol{Q}})$ is close to zero, the estimate of π_m will become close to zero if we use $Dir(1, ..., 1)$. This will make the algorithm numerically unstable since the EM is implemented at logarithm scale (i.e., $\ln(\pi_m)$ instead of π_m is used in the implementation to avoid underflow when multiplying multiple probabilities). The same reason explains why $B(2, 2)$ was chosen as the prior for q_{kd}.

A.3. The EM algorithm used in cormotif

This section presents the EM algorithm used to search for posterior mode of $\hat{\boldsymbol{\pi}}$ and $\hat{\boldsymbol{Q}}$ of the distribution $Pr(\boldsymbol{\pi}, \boldsymbol{Q}|\boldsymbol{T}) = \sum_{\boldsymbol{A},\boldsymbol{B}} Pr(\boldsymbol{\pi}, \boldsymbol{Q}, \boldsymbol{A}, \boldsymbol{B}|\boldsymbol{T})$. In the EM algorithm, $\boldsymbol{A}$ and $\boldsymbol{B}$ are missing data. The algorithm iterates between an E-step and an M-step.

In the E-step, one evaluates the Q-function $Q(\boldsymbol{\pi}, \boldsymbol{Q}|\hat{\boldsymbol{\pi}}^{\boldsymbol{old}}, \hat{\boldsymbol{Q}}^{\boldsymbol{old}})$, defined as $E_{old}[\ln Pr(\boldsymbol{\pi}, \boldsymbol{Q}, \boldsymbol{A}, \boldsymbol{B}|\boldsymbol{T})]$. Here the expectation is taken with respect to distribution $Pr(\boldsymbol{A}, \boldsymbol{B}|\boldsymbol{T}, \hat{\boldsymbol{\pi}}^{\boldsymbol{old}}, \hat{\boldsymbol{Q}}^{\boldsymbol{old}})$, abbreviated as $Pr_{old}(\boldsymbol{A}, \boldsymbol{B})$, where $\hat{\boldsymbol{\pi}}^{\boldsymbol{old}}$ and $\hat{\boldsymbol{Q}}^{\boldsymbol{old}}$ are the parameter estimates obtained from the last iteration.

We have

$$\ln Pr(\boldsymbol{\pi}, \boldsymbol{Q}, \boldsymbol{A}, \boldsymbol{B}|\boldsymbol{T}) = \sum_{g=1}^{G}\sum_{k=1}^{K}\delta(b_g = k)\ln \pi_k$$
$$+\sum_{g=1}^{G}\sum_{k=1}^{K}\delta(b_g = k)\left\{\sum_{d=1}^{D}a_{gd}[\ln q_{kd} + \ln f_{d1}(x_{gd})] + \sum_{d=1}^{D}(1-a_{gd})[\ln(1-q_{kd}) + \ln f_{d0}(x_{gd})]\right\}$$
$$+\sum_{k=1}^{K}\ln \pi_k + \sum_{k=1}^{K}\sum_{d=1}^{D}[\ln q_{kd} + \ln(1-q_{kd})] + constant \tag{A.1}$$

Therefore,

$$Q(\boldsymbol{\pi}, \boldsymbol{Q}|\hat{\boldsymbol{\pi}}^{\boldsymbol{old}}, \hat{\boldsymbol{Q}}^{\boldsymbol{old}}) = E_{old}[\ln Pr(\boldsymbol{\pi}, \boldsymbol{Q}, \boldsymbol{A}, \boldsymbol{B}|\boldsymbol{T})]$$
$$= \sum_{g=1}^{G}\sum_{k=1}^{K}\ln \pi_k E_{old}(\delta(b_g = k))$$
$$+\sum_{g=1}^{G}\sum_{k=1}^{K}\sum_{d=1}^{D}[\ln q_{kd} + \ln f_{d1}(x_{gd})]E_{old}(\delta(b_g = k)a_{gd})$$
$$+\sum_{g=1}^{G}\sum_{k=1}^{K}\sum_{d=1}^{D}[\ln(1-q_{kd}) + \ln f_{d0}(x_{gd})]E_{old}(\delta(b_g = k)(1-a_{gd}))$$
$$+\sum_{k=1}^{K}\ln \pi_k + \sum_{k=1}^{K}\sum_{d=1}^{D}[\ln q_{kd} + \ln(1-q_{kd})] + constant \tag{A.2}$$

In the M-step, one finds $\boldsymbol{\pi}$ and $\boldsymbol{Q}$ that maximize the Q-function $Q(\boldsymbol{\pi}, \boldsymbol{Q}|\hat{\boldsymbol{\pi}}^{\boldsymbol{old}}, \hat{\boldsymbol{Q}}^{\boldsymbol{old}})$. Denote them as $\hat{\boldsymbol{\pi}}^{\boldsymbol{new}}$ and $\hat{\boldsymbol{Q}}^{\boldsymbol{new}}$. They will be used in the next iteration.

By solving

$$\frac{\partial Q(\boldsymbol{\pi}, \boldsymbol{Q}|\hat{\boldsymbol{\pi}}^{\boldsymbol{old}}, \hat{\boldsymbol{Q}}^{\boldsymbol{old}})}{\partial \pi_k} = 0 \tag{A.3}$$

$$\frac{\partial Q(\boldsymbol{\pi}, \boldsymbol{Q}|\hat{\boldsymbol{\pi}}^{\boldsymbol{old}}, \hat{\boldsymbol{Q}}^{\boldsymbol{old}})}{\partial q_{kd}} = 0 \tag{A.4}$$

We have

$$\hat{\pi}_k^{new} = \frac{\sum_{g=1}^{G} Pr_{old}(b_g = k) + 1}{G + K} \tag{A.5}$$

$$\hat{q}_{kd}^{new} = \frac{\sum_{g=1}^{G} Pr_{old}(b_g = k, a_{gd} = 1) + 1}{\sum_{g=1}^{G} Pr_{old}(b_g = k) + 2} \tag{A.6}$$

In the formulae above, $Pr_{old}(b_g = k)$ and $Pr_{old}(b_g = k, a_{gd} = 1)$ can be computed as below

$$Pr_{old}(b_g = k) = \frac{\hat{\pi}_k^{(old)} \prod_{d=1}^{D} [\hat{q}_{kd}^{(old)} f_{d1}(t_{gd}) + (1 - \hat{q}_{kd}^{(old)}) f_{d0}(t_{gd})]}{\sum_{l=1}^{K} \hat{\pi}_l^{(old)} \prod_{d=1}^{D} [\hat{q}_{ld}^{(old)} f_{d1}(t_{gd}) + (1 - \hat{q}_{ld}^{(old)}) f_{d0}(t_{gd})]} \tag{A.7}$$

$$\begin{aligned} Pr_{old}(b_g = k, a_{gd} = 1) &= Pr_{old}(a_{gd} = 1 | b_g = k) * Pr_{old}(b_g = k) \\ &= \frac{\hat{q}_{kd}^{(old)} f_{d1}(t_{gd})}{\hat{q}_{kd}^{(old)} f_{d1}(t_{gd}) + (1 - \hat{q}_{kd}^{(old)}) f_{d0}(t_{gd})} Pr_{old}(b_g = k) \end{aligned} \tag{A.8}$$

The E-step and M-step will iterate until convergence. The algorithm stops when none of the parameters in $\boldsymbol{\pi}$ and $\boldsymbol{Q}$ changes by more than 0.1% compared to their values in the previous iteration. Using this algorithm, we can obtain estimates for $\boldsymbol{\pi}$ and $\boldsymbol{Q}$.

A.4. Bayesian information criterion (BIC) and algorithm for choosing K

BIC is computed as

$$\begin{aligned} BIC(K) &= -2 * \ln Pr(\boldsymbol{T} | \boldsymbol{\pi}, \boldsymbol{Q}) + (K - 1 + K * D) * \ln G \\ &= -2 * \sum_{g=1}^{G} \ln \left[\sum_{k=1}^{K} \{ \pi_k \prod_{d=1}^{D} [q_{kd} f_{d1}(t_{gd}) + (1 - q_{kd}) f_{d0}(t_{gd})] \} \right] + (K - 1 + K * D) * \ln G \end{aligned} \tag{A.9}$$

Here K is the number of motifs in the data. $K - 1$ is the number of parameters for $\boldsymbol{\pi}$. KD is the number of parameters involved in $\boldsymbol{Q}$. G is the gene number.

In order to choose K, BIC for different values of K are calculated. The K corresponding to the smallest BIC is chosen. Intuitively, to implement this, one can start with $K = 1$. After evaluating BIC at $K = 1$, one will increase K by one and evaluates the BIC again. This procedure will be

repeated until one finds a K such that $BIC(K) < BIC(K+1)$ and $BIC(K) < BIC(K+2)$ and $BIC(K) < BIC(K')$ for all $K' < K$, at which point the algorithm will stop and the K will be reported as the final motif number.

The algorithm above does not impose any upper limit on K. If the optimal K is big, it may require one to compute BIC for many different Ks which could make the computation slow. In order to make the computation faster, *CorMotif* actually uses a modified algorithm as follows.

1. Set a start point $K_0 \leftarrow 1$ and a step size s. The initial step size can be relatively big (e.g., $s = 10$) and is set by users.

2. Start with $K = K_0$. After evaluating BIC at $K = K_0$, increase K by s (i.e., $K \leftarrow K + s$) and evaluate the BIC again. This procedure will be repeated until one finds a K such that $BIC(K) < BIC(K+s)$ and $BIC(K) < BIC(K+2s)$ and $BIC(K) < BIC(K')$ for all $K' < K$ (note: here $K - K'$ is a multiple of the step size s). This K will be recorded and denoted as K_m.

3. If the step size $s = 1$, then report K_m as the optimal K and exit the algorithm. Otherwise, the optimal K should be between $K_m - s$ and $K_m + s$. One can search it within this range using a smaller step size. To do so, reset the start point $K_0 \leftarrow \max(K_m - s, 1)$, and reset the step size $s \leftarrow \lfloor s/2 \rfloor$. Here $\lfloor . \rfloor$ returns the largest integer that does not exceed $s/2$. Go back to step 2.

Again, this algorithm does not impose any upper limit for the motif number K. We note, however, that in real applications, we seldom see cases where the optimal K is big. Based on our own experience, the optimal K often is smaller than 10.

In all simulations and real data analyses in this article, *CorMotif* was run using $s = 1$, and the optimal K with the minimal BIC was all achieved below 10.

A.5. Data for real data based simulations

Simulations 5-10 were based on real data characteristics. Each simulation contained multiple studies, and each study was composed of six samples from the same GEO experiment with the same biological condition as detailed in Table A.4. The six samples were further split into three pseudo cases and three pseudo controls. They were used as the simulated background since one does not expect any real differential signals between replicate samples. We then spiked in differential signals by adding random $N(0, 1)$ numbers to the three cases according to the patterns shown in Figures A.2 (a,d,g) and A.4(a-b,e-f,i-j,m-n). Data simulated in this way were able to keep the background characteristics in real data.

A.6. Discussion on the two-stage design

Currently, *CorMotif* is based on modeling the moderated t-statistics t_{gd}. Instead of using this two-stage approach, a potential future extension is to introduce a single coherent Bayesian model that fully integrates the correlation motifs with a model directly describing the raw expression values x_{gdlj}. In the present study, we chose to use the two-stage approach for several reasons.

First, it allows us to better present the core idea of this paper, that is, how to use correlation motifs to integrate multiple studies. By taking advantage of the well-documented *limma* approach, the two-stage approach allows us to simplify the presentation of some of the model details (i.e., those related to the moderated t-statistics) and use the limited amount of available space in the main article to focus on discussing the core idea of correlation motifs. Although a more coherent and sophisticated model for x_{gdlj} may bring some additional performance gain, the burden for us to present and for readers to digest additional notations and model details may distract one from focusing on the core part of our approach. The space limit forces us to find a trade-off between these two. We believe that it is more important for one to understand the correlation motif idea. Once one gets this idea, one can easily improve it by extending the data models. Moreover, the

two-stage approach as presented now also represents a very general framework. Conceptually, one can modify f_{d0} and f_{d1} to accommodate other data types. Because of the two-stage design, this will not change the correlation motif model and the corresponding EM algorithm.

Second, using the two-stage framework, one can develop a simple EM algorithm to fit the model. This approach is computationally more efficient than running a Markov Chain Monte Carlo (MCMC) algorithm on a fully Bayesian model with many levels of unknown parameters (e.g., mean and variances of x_{gdlj}s and parameters in their prior distributions, missing indicators $\boldsymbol{A}$ and $\boldsymbol{B}$, and motif parameters $\boldsymbol{\pi}$ and $\boldsymbol{Q}$).

Third, the present design also allowed us to perform a well-controlled comparison with the state-of-the-art approach *limma*. In our two-stage design, the first stage of *CorMotif* uses the same model as *limma* to compute the moderated t-statistics. The only difference between *CorMotif* and *limma* is in the second stage, that is, the correlation motif part. For this reason, the comparison between *CorMotif* and *limma* can unambiguously demonstrate the gain of using correlation motifs to integrate multiple studies. This gain is not confounded with other factors such as differences in the data distributions f_{d0} and f_{d1}. By contrast, differences in performance between *CorMotif* and other methods such as *SAM* and *eb1*, etc., can be caused by a number of different factors such as differences in models for data x_{gdlj}. The two-stage design therefore has helped us to perform a clean comparison to show the effectiveness of correlation motifs. As a result, we were able to contribute a general tool with proven effectiveness (i.e., the correlation motif framework for data integration) to the toolbox other people can use to build future data analysis methods.

A.7. Discussion on computational time

We compared the computation time of different algorithms. We used real data based simulations 5-7 (with study number $D = 4$, 8 and 20 respectively) as well as the real SHH data to do this comparison in order to provide a realistic picture. All algorithms were run in a single 2.7GHz

CPU with 4Gb RAM. The results are shown in Table A.9. The computation time shown for *CorMotif* and *eb10best* includes the time used for searching the optimal motif number K (see Section A.4 for the algorithm used by *CorMotif* to search for K). For these two algorithms, the model was fitted at multiple different K values, and the average computation time for each K (i.e., the mean time required for a single K, also called "per K time") is also shown as "CorMotif (mean)" and "eb10best (mean)". The other methods do not need to search for K.

Based on the results, the total computation time required by *CorMotif* was between *eb1* and *eb10best* when the study number D is relatively small (i.e., simulation 5 and SHH data). However, both *eb1* and *eb10best* became very slow or failed to run when D became big (i.e., simulations 6 and 7). *CorMotif*, *eb1* and *eb10best* were all slower than *SAM* and *all concord*. *SAM* analyzes each study separately and does not involve computation-intensive iterations. *All concord* assumes concordant signals and usually converges in a few iterations. These explain why they were fast. *Separate limma* also analyzes each study separately. However, recall that in this article an iterative EM algorithm was added to *separate limma* to call differential expression in order to match with *CorMotif* to better evaluate the gain in statistical power brought by correlation motifs. For this reason, *separate limma* was much slower than *SAM* in some datasets (e.g., simulation 7 and the real SHH data) because the EM algorithm took more iterations to converge in those datasets. In fact, when we used the original *limma* without adding the EM algorithm, the computation time was reduced to the same level as *SAM* (see "limma (original)" in Table A.9). When the study number D is small, the number of unknown parameters for *CorMotif* is comparable to or may even be bigger than that of *full motif*. For instance, if the study number $D = 4$ and the motif number $K = 4$, the number of parameters in $\boldsymbol{\pi}$ and $\boldsymbol{Q}$ in *CorMotif* is $K - 1 + K * D = 4 - 1 + 4 * 4 = 19$, whereas the number of equivalent parameters in *full motif* is $2^D = 2^4 = 16 < 19$. This and many other factors including the number of iterations to convergence and implementation details may all affect the computation time. Consistent with

this, the average computation time required by *CorMotif* for a single K (i.e., the per K time) was longer than that for *full motif* in simulation 5. In simulation 6 and real SHH data, their per K computation time became comparable. However, in simulation 7 where D was big, *full motif* failed to run. One additional thing to note is that the computation time is also affected by the signal-to-noise ratio. For example, the per K time for *CorMotif* in simulation 7 which involved more studies is slightly smaller than that in simulation 6 which involved fewer studies. This was because with signals integrated from 20 studies, the signal-to-noise ratio in simulation 7 was stronger, leading to a faster convergence. Together, our results show that *CorMotif* is computationally tractable and it is able to handle a large number of studies without having the exponential complexity problem. We also note that *CorMotif* has a parameter that allows users to fit the model using a fixed and user-specified motif number K. Therefore, if one has multiple processors, the computation could be accelerated by running the model fitting jobs for different Ks in parallel in different CPUs.

References

Jensen, S.T., Erkan, I., Arnardottir, E.S. and Small, D.S. (2009). Bayesian testing of many hypothesis*many genes: a study of sleep apnea. *Annals of Applied Statistics* **3(3)**, 1080–1101.

Kendziorski, C.M., M.A. Newton, M. A.and H. Lan and Gould, M.N. (2003). On parametric empirical bayes methods for comparing multiple groups using replicated gene expression profiles. *Statistics in Medicine* **22**, 3899–3914.

Ruan, L. and Yuan, M. (2011). An empirical bayes approach to joint analysis of multiple microarray gene expression studies. *Biometrics* **67**, 1617C–1626.

Scharpf, R.B., Tjelmeland, H., Parmigiani, G. and Nobel, A.B. (2009). A bayesian

model for cross-study differential gene expression. *Journal of the American Statistical Association* **104(488)**, 1295–1310.

Yuan, M. and Kendziorski, C.M. (2006). A unified approach for simultaneous gene clustering and differential expression identification. *Biometrics* **62**, 1089–1098.

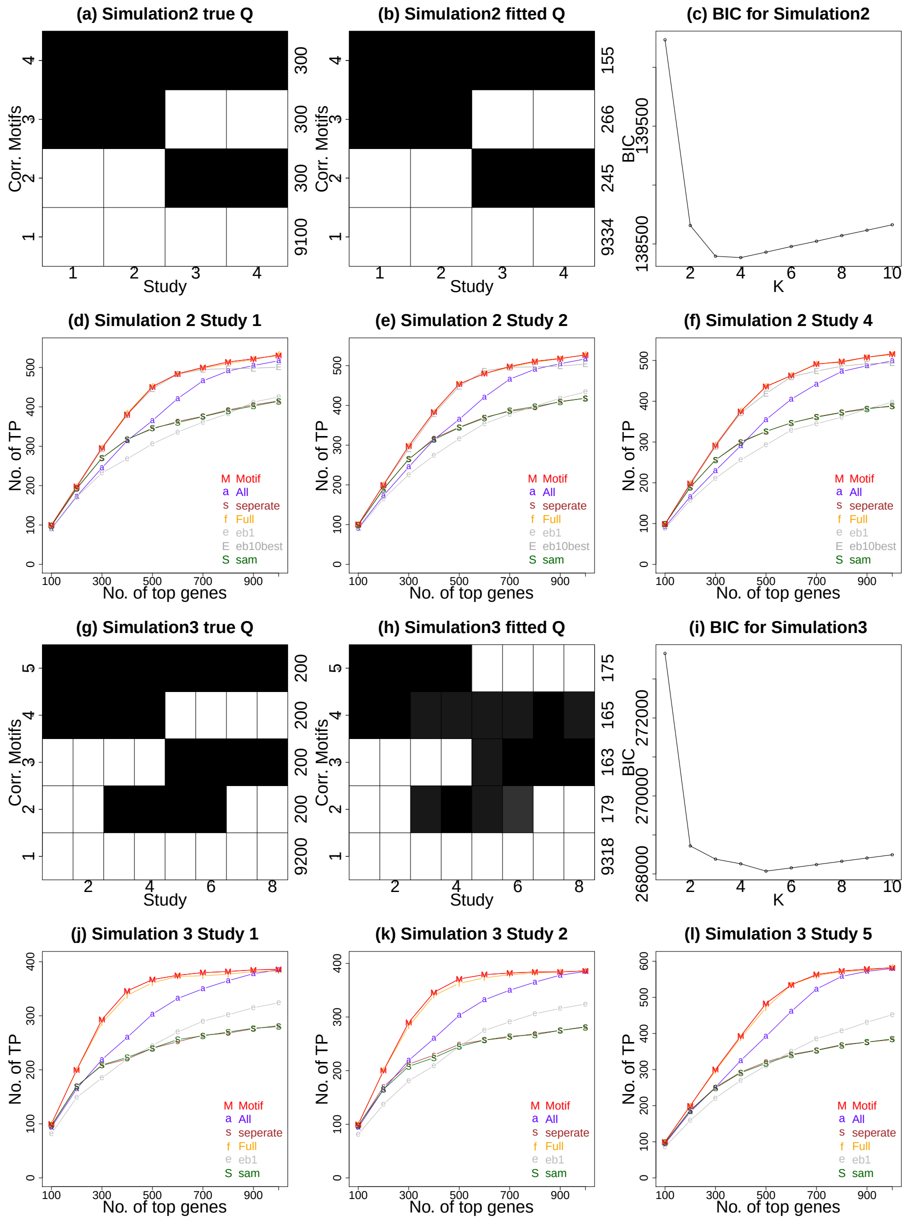

Fig. A.1. Results for the model assumption based simulations 2 and 3. (a) and (g) Motif patterns for simulations 2 and 3. The $\boldsymbol{Q}$ of the true motifs is shown. Each row indicates a motif pattern and each column represents a study. The actual number of genes belonging to each motif (i.e., $\boldsymbol{\pi} * G$) is displayed at the right end of each row. The gray scale of the cell (k, d) demonstrates the probability of differential expression in study d for pattern k. Black means 1 and white means 0. (b) and (h) The estimated $\hat{\boldsymbol{Q}}$ from the learned motifs with $\hat{\boldsymbol{\pi}} * G$ annotated at the end of each row. (c) and (i) BIC plots. It can be seen that motif patterns reported by *CorMotif* under the minimal BIC are similar to the true underlying motif patterns. (d)-(f) and (j)-(l) Gene ranking performance of different methods in simulations 2 and 3. $TP_d(r)$, the number of genes that are truly differentially expressed in study d among the top r ranked genes by a given method, is plotted against the rank cutoff r. For each simulation, results for a few representative studies are shown. Each plot is for one study.

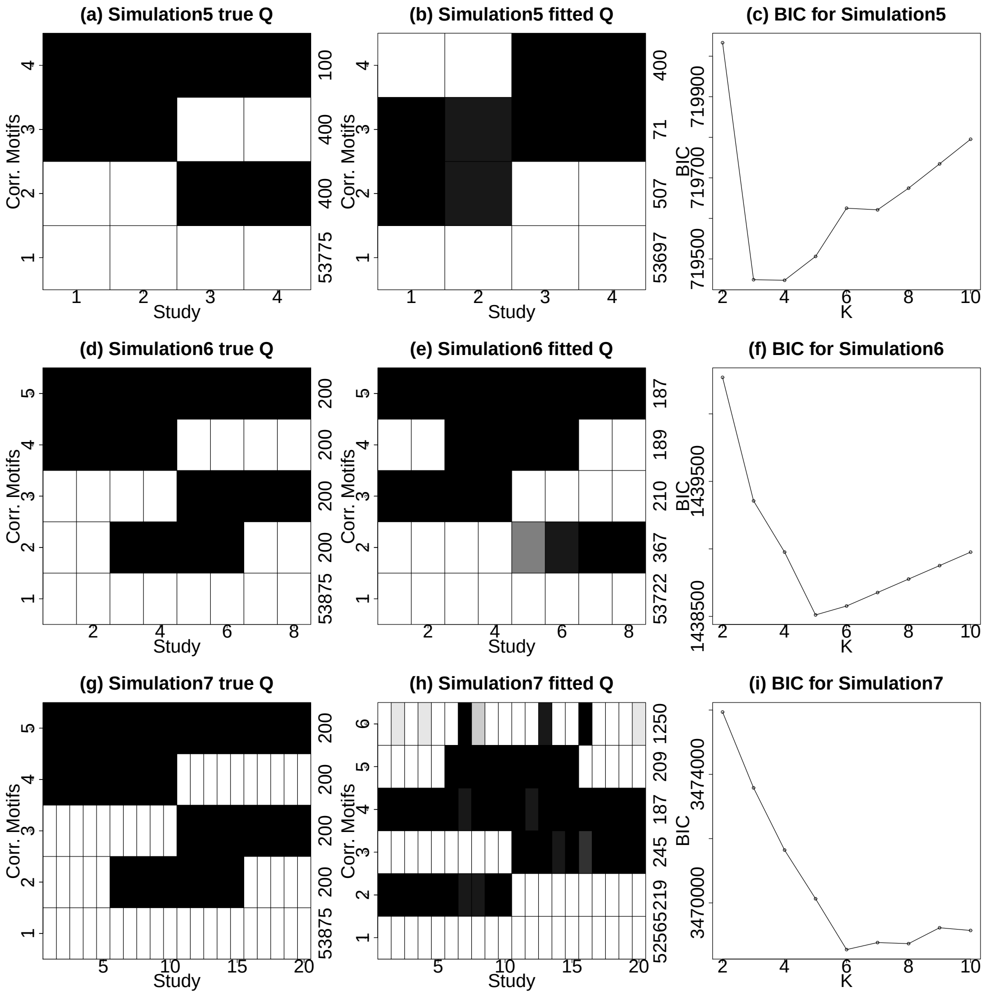

Fig. A.2. Motif patterns for simulations 5, 6 and 7. (a)(d)(g) The $\boldsymbol{Q}$ of the true motifs is shown. Each row indicates a motif pattern and each column represents a study. The actual number of genes belonging to each motif (i.e., $\boldsymbol{\pi} * G$) is displayed at the right end of each row. The gray scale of the cell (k, d) demonstrates the probability of differential expression in study d for pattern k. Black means 1 and white means 0. (b)(e)(h) The estimated $\hat{\boldsymbol{Q}}$ from the learned motifs with $\hat{\boldsymbol{\pi}} * G$ annotated at the end of each row. (c)(f)(i) BIC plots.

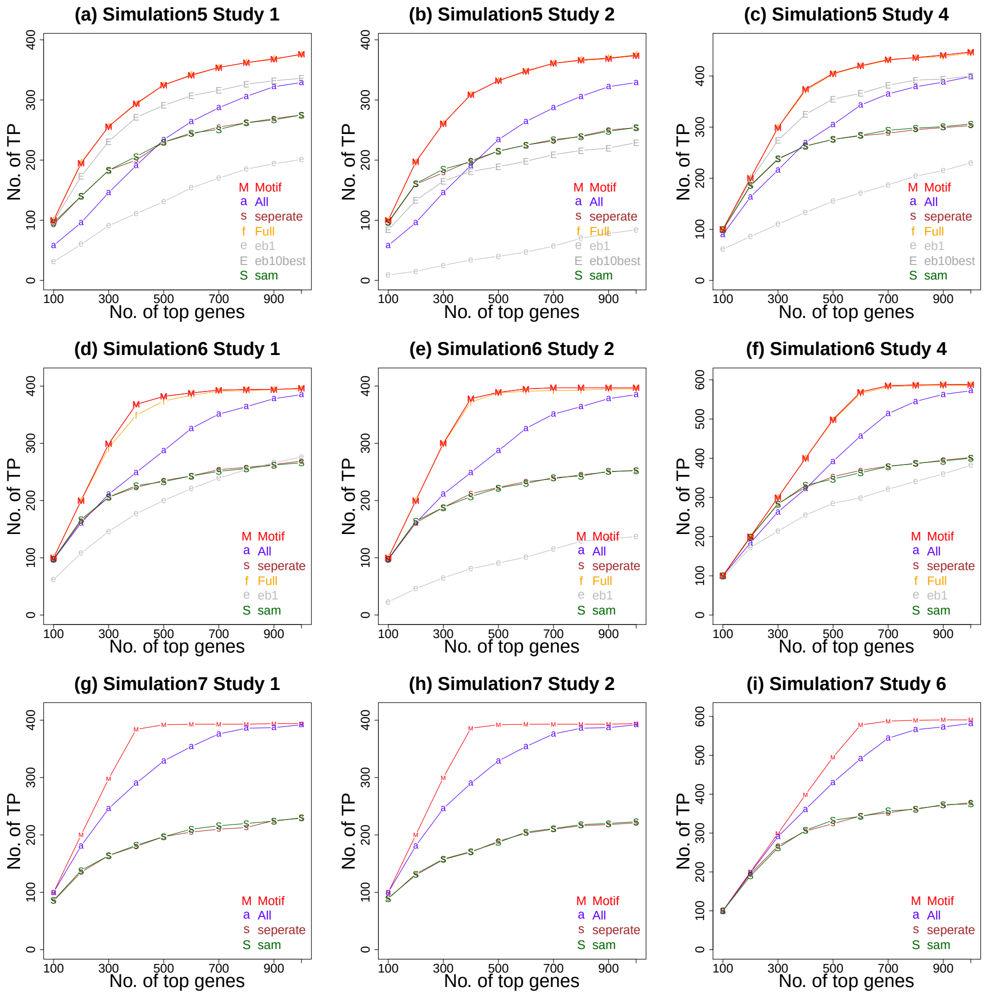

Fig. A.3. Gene ranking performance for simulations 5, 6 and 7. $TP_d(r)$, the number of genes that are truly differentially expressed in study d among the top r ranked genes by a given method, is plotted against the rank cutoff r. For each simulation, results for a few representative studies are shown. Each plot is for one study. (a)-(c) Gene ranking performance for simulation 5. (d)-(f) Gene ranking performance for simulation 6. (g)-(i) Gene ranking performance for simulation 7.

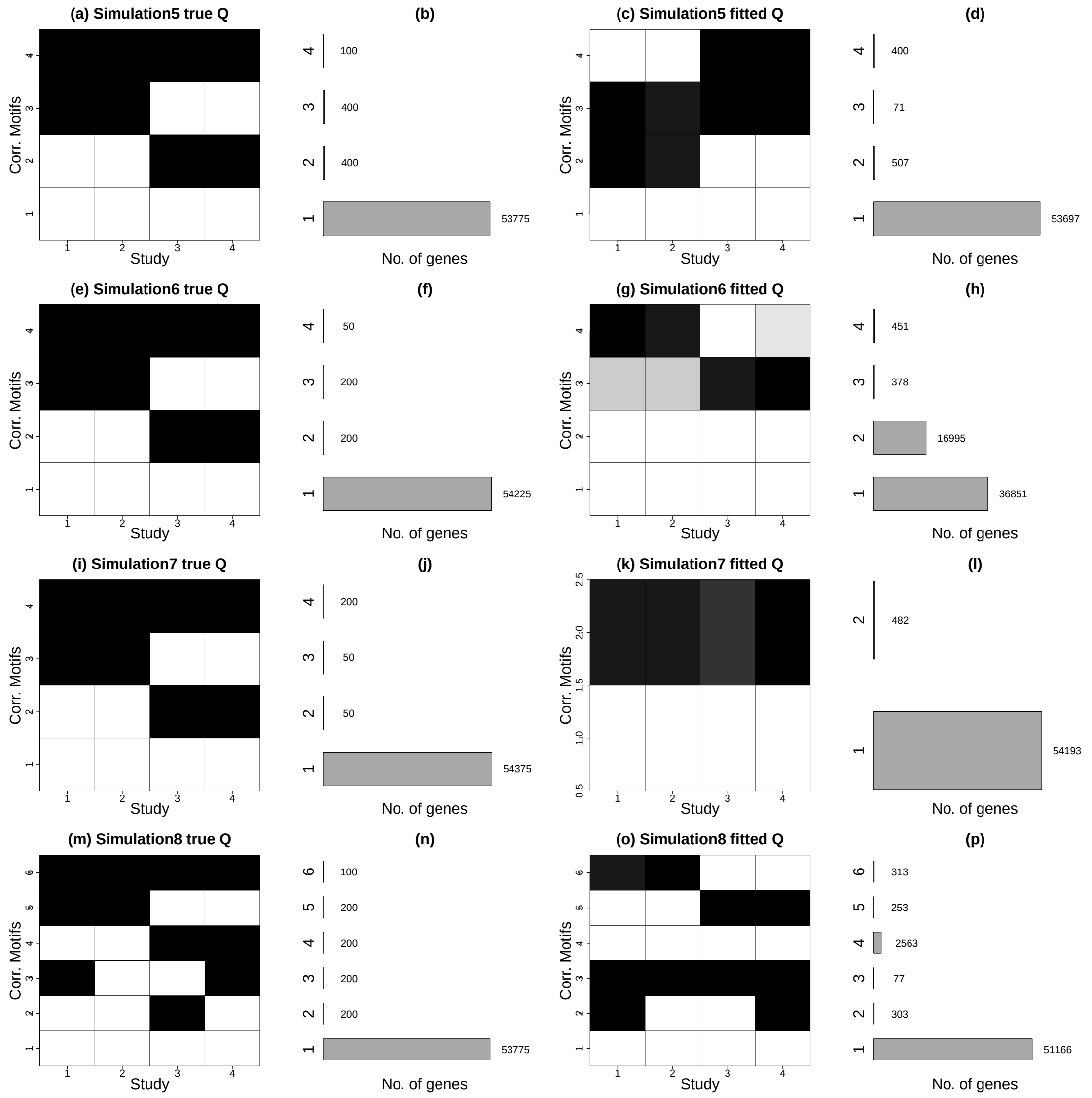

Fig. A.4. Motif patterns for simulations 5, 8, 9 and 10. (a),(e),(i),(m) The $\boldsymbol{Q}$ for the true underlying motifs in the simulated data. (b),(f),(j),(n) The true number of genes belonging to each motif in the simulated data (i.e., $\boldsymbol{\pi} * G$). (c),(g),(k),(o) The estimated $\hat{\boldsymbol{Q}}$ for the learned motifs. (d),(h),(l),(p) The estimated number of genes belonging to each learned motif (i.e., $\hat{\boldsymbol{\pi}} * G$). In the $\boldsymbol{Q}$ pattern graph (columns 1 and 3), each row indicates a motif pattern and each column represents a study. The gray scale of the cell (k, d) demonstrates the probability of differential expression in study d for pattern k. Each row of the bar chart for $(\boldsymbol{\pi} * G)$ corresponds to the motif pattern in the same row of the $\boldsymbol{Q}$ graph. The motif patterns learned by *CorMotif* are similar to the true underlying motif patterns. It can be seen that complementary block motifs, such as [1,1,0,0] and [0,0,1,1], are not likely to be absorbed into merged motifs if their relative proportions are not low.

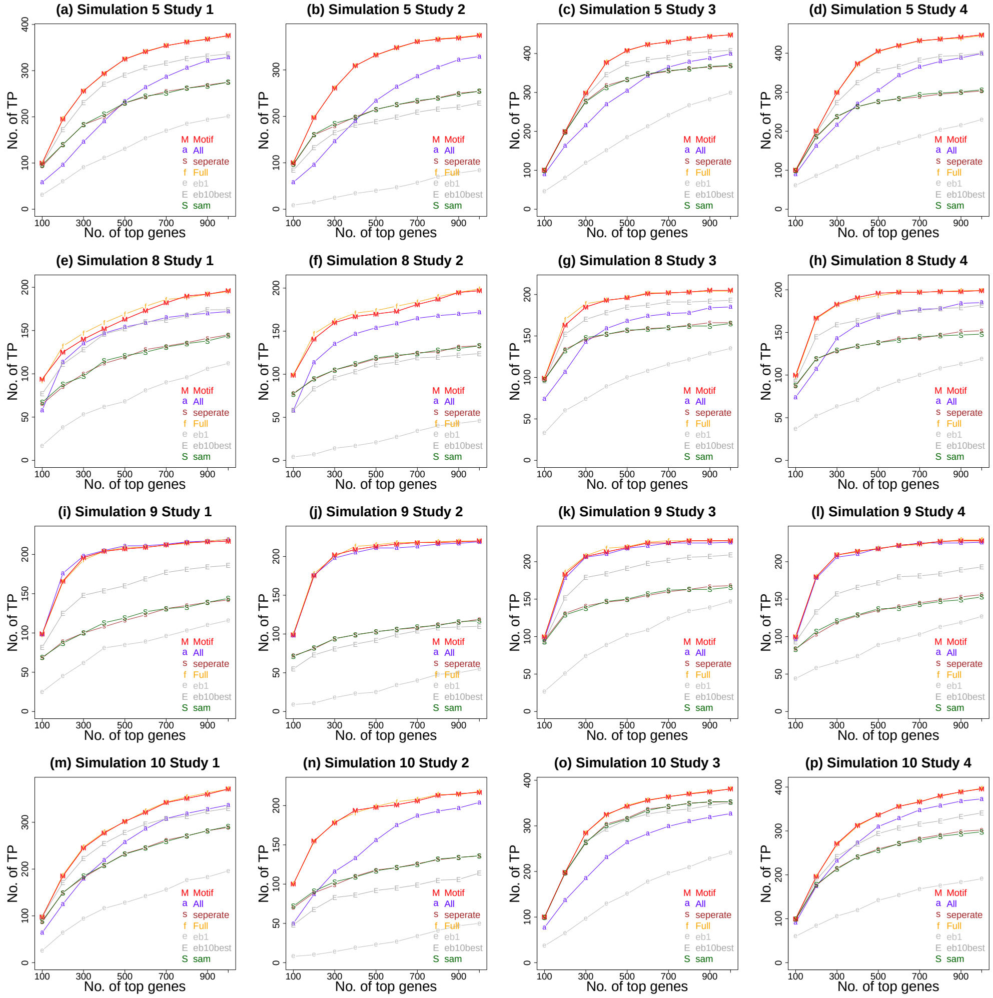

Fig. A.5. Gene ranking performance for simulations 5, 8, 9 and 10. $TP_d(r)$, the number of genes that are truly differentially expressed in study d among the top r ranked genes by a given method, is plotted against the rank cutoff r. (a)-(d) Simulation 5. (e)-(h) Simulation 8. (i)-(l) Simulation 9. (m)-(p) Simulation 10.

Table A.1. Confusion matrix for simulation 2. The column labels indicate the true underlying patterns and the row labels represent the learned configurations.

Method	Differential configuration	$c(0,0,0,0)$	$c(0,0,1,1)$	$c(1,1,0,0)$	$c(1,1,1,1)$
Cormotif	$c(0,0,0,0)$	9069	122	99	54
	$c(0,0,1,1)$	7	127	0	30
	$c(1,1,0,0)$	3	0	153	29
	$c(1,1,1,1)$	0	1	1	89
	other	21	50	47	98
separate limma	$c(0,0,0,0)$	9024	112	89	58
	$c(0,0,1,1)$	1	44	0	13
	$c(1,1,0,0)$	0	0	57	17
	$c(1,1,1,1)$	0	0	0	8
	other	75	144	154	204
all concord	$c(0,0,0,0)$	9094	180	166	76
	$c(0,0,1,1)$	0	0	0	0
	$c(1,1,0,0)$	0	0	0	0
	$c(1,1,1,1)$	6	120	134	224
	other	0	0	0	0
full motif	$c(0,0,0,0)$	9069	122	99	54
	$c(0,0,1,1)$	7	130	0	33
	$c(1,1,0,0)$	5	0	160	29
	$c(1,1,1,1)$	0	1	1	99
	other	19	47	40	85
eb1	$c(0,0,0,0)$	4693	20	8	5
	$c(0,0,1,1)$	376	65	1	8
	$c(1,1,0,0)$	474	1	74	10
	$c(1,1,1,1)$	365	131	132	238
	other	3192	83	85	39
eb10best	$c(0,0,0,0)$	0	0	0	0
	$c(0,0,1,1)$	79	188	1	30
	$c(1,1,0,0)$	68	0	202	31
	$c(1,1,1,1)$	7793	105	87	223
	other	1160	7	10	16
SAM	$c(0,0,0,0)$	9095	209	236	193
	$c(0,0,1,1)$	0	7	0	6
	$c(1,1,0,0)$	0	0	0	0
	$c(1,1,1,1)$	0	0	0	0
	other	5	84	64	101

Table A.2. Confusion matrix for simulation 3. The column labels indicate the true underlying patterns and the row labels represent the learned configurations.

Method	Differential configuration	$Motif1$	$Motif2$	$Motif3$	$Motif4$	$Motif5$
CorMotif	$Motif1$	9189	28	48	50	4
	$Motif2$	0	68	0	0	4
	$Motif3$	0	1	65	0	5
	$Motif4$	0	2	0	97	6
	$Motif5$	0	0	0	0	27
	other	11	101	87	53	154
separate limma	$Motif1$	9076	24	36	43	3
	$Motif2$	0	2	0	0	0
	$Motif3$	0	0	2	0	0
	$Motif4$	0	0	0	3	1
	$Motif5$	0	0	0	0	0
	other	124	174	162	154	196
all concord	$Motif1$	9200	96	117	94	5
	$Motif2$	0	0	0	0	0
	$Motif3$	0	0	0	0	0
	$Motif4$	0	0	0	0	0
	$Motif5$	0	104	83	106	195
	other	0	0	0	0	0
full motif	$Motif1$	9185	28	46	49	4
	$Motif2$	0	63	0	0	3
	$Motif3$	0	0	51	0	4
	$Motif4$	0	2	0	89	3
	$Motif5$	0	0	0	0	14
	other	15	107	103	62	172
eb1	$Motif1$	748	0	1	1	0
	$Motif2$	273	2	0	0	0
	$Motif3$	4	0	1	0	0
	$Motif4$	47	0	0	0	0
	$Motif5$	1239	157	149	170	183
	other	6889	41	49	29	17
SAM	$Motif1$	9200	139	170	165	134
	$Motif2$	0	0	0	0	0
	$Motif3$	0	0	0	0	0
	$Motif4$	0	0	0	0	0
	$Motif5$	0	0	0	0	0
	other	0	61	30	35	66

Table A.3. Confusion matrix for simulation 4. The column labels indicate the true underlying patterns and the row labels represent the learned configurations.

Method	Differential configuration	$Motif1$	$Motif2$	$Motif3$	$Motif4$	$Motif5$
CorMotif	$Motif1$	9198	4	5	2	0
	$Motif2$	0	29	0	0	0
	$Motif3$	0	0	20	0	0
	$Motif4$	0	0	0	22	0
	$Motif5$	0	0	0	0	4
	other	2	167	175	176	196
separate limma	$Motif1$	8907	1	3	1	0
	$Motif2$	0	0	0	0	0
	$Motif3$	0	0	0	0	0
	$Motif4$	0	0	0	0	0
	$Motif5$	0	0	0	0	0
	other	293	199	197	199	200
all concord	$Motif1$	9200	58	69	69	0
	$Motif2$	0	0	0	0	0
	$Motif3$	0	0	0	0	0
	$Motif4$	0	0	0	0	0
	$Motif5$	0	142	131	131	200
	other	0	0	0	0	0
SAM	$Motif1$	9197	64	66	92	23
	$Motif2$	0	0	0	0	0
	$Motif3$	0	0	0	0	0
	$Motif4$	0	0	0	0	0
	$Motif5$	0	0	0	0	0
	other	3	136	134	108	177

Table A.4. GEO data used for real data based simulations.

Simulation ID	Study ID	GEO Sample Id	GEO series number	Sample No.	Sample type
Simulations 5-10	1	GSM366065.CEL - GSM366070.CEL	GSE14668	6	Liver tissue of liver donor
Simulations 5-10	2	GSM550623.CEL - GSM550628.CEL	GSE22138	6	Uveal Melanoma primary tumor tissue
Simulations 5-10	3	GSM553482.CEL - GSM553487.CEL	GSE22224	6	Peripheral blood mononuclear cells of healthy volunteer
Simulations 5-10	4	GSM494634.CEL - GSM494639.CEL	GSE33356	6	Normal lung tissue
Simulations 6-7	5	GSM909644.CEL - GSM909649.CEL	GSE37069	6	Blood samples from controls
Simulations 6-7	6	GSM909650.CEL - GSM909655.CEL	GSE37069	6	Blood samples from controls
Simulations 6-7	7	GSM909656.CEL - GSM909661.CEL	GSE37069	6	Blood samples from controls
Simulations 6-7	8	GSM909662.CEL - GSM909667.CEL	GSE37069	6	Blood samples from controls
Simulations 6-7	9	GSM90968.CEL - GSM909673.CEL	GSE37069	6	Blood samples from controls
Simulations 6-7	10	GSM909674.CEL - GSM909679.CEL	GSE37069	6	Blood samples from controls
Simulation 7	11	GSM376428.CEL - GSM376433.CEL	GSE15061	6	Non-leukemia bone marrow samples
Simulation 7	12	GSM376434.CEL - GSM376439.CEL	GSE15061	6	Non-leukemia bone marrow samples
Simulation 7	13	GSM376440.CEL - GSM376445.CEL	GSE15061	6	Non-leukemia bone marrow samples
Simulation 7	14	GSM376446.CEL - GSM376451.CEL	GSE15061	6	Non-leukemia bone marrow samples
Simulation 7	15	GSM376452.CEL - GSM376457.CEL	GSE15061	6	Non-leukemia bone marrow samples
Simulation 7	16	GSM376458.CEL - GSM376463.CEL	GSE15061	6	Non-leukemia bone marrow samples
Simulation 7	17	GSM376464.CEL - GSM376469.CEL	GSE15061	6	Non-leukemia bone marrow samples
Simulation 7	18	GSM376470.CEL - GSM376475.CEL	GSE15061	6	Non-leukemia bone marrow samples
Simulation 7	19	GSM376476.CEL - GSM376481.CEL	GSE15061	6	Non-leukemia bone marrow samples
Simulation 7	20	GSM376482.CEL - GSM376487.CEL	GSE15061	6	Non-leukemia bone marrow samples

Table A.5. Confusion matrix for simulation 5. The column labels indicate the true underlying patterns and the row labels represent the learned configurations.

Method	Differential configuration	$c(0,0,0,0)$	$c(0,0,1,1)$	$c(1,1,0,0)$	$c(1,1,1,1)$
CorMotif	$c(0,0,0,0)$	53670	108	164	20
	$c(0,0,1,1)$	6	286	0	18
	$c(1,1,0,0)$	29	0	200	6
	$c(1,1,1,1)$	0	0	0	31
	other	70	6	36	25
separate limma	$c(0,0,0,0)$	53615	121	171	24
	$c(0,0,1,1)$	0	79	0	8
	$c(1,1,0,0)$	0	0	46	3
	$c(1,1,1,1)$	0	0	0	1
	other	160	200	183	64
all concord	$c(0,0,0,0)$	53748	187	255	26
	$c(0,0,1,1)$	0	0	0	0
	$c(1,1,0,0)$	0	0	0	0
	$c(1,1,1,1)$	27	213	145	74
	other	0	0	0	0
full motif	$c(0,0,0,0)$	53671	108	165	20
	$c(0,0,1,1)$	5	286	0	18
	$c(1,1,0,0)$	30	0	201	6
	$c(1,1,1,1)$	0	0	1	36
	other	69	6	33	20
eb1	$c(0,0,0,0)$	49817	190	188	23
	$c(0,0,1,1)$	161	103	0	12
	$c(1,1,0,0)$	244	0	66	8
	$c(1,1,1,1)$	11	0	0	7
	other	3542	107	146	50
eb10best	$c(0,0,0,0)$	51731	109	125	36
	$c(0,0,1,1)$	5	232	0	6
	$c(1,1,0,0)$	12	0	169	4
	$c(1,1,1,1)$	0	0	0	16
	other	2027	59	106	38
SAM	$c(0,0,0,0)$	53773	283	398	83
	$c(0,0,1,1)$	0	0	0	0
	$c(1,1,0,0)$	0	0	0	0
	$c(1,1,1,1)$	0	0	0	0
	other	2	117	2	17

Table A.6. Confusion matrix for simulation 6. The column labels indicate the true underlying patterns and the row labels represent the learned configurations.

Method	Differential configuration	$Motif1$	$Motif2$	$Motif3$	$Motif4$	$Motif5$
CorMotif	$Motif1$	53600	15	11	15	1
	$Motif2$	0	169	0	1	4
	$Motif3$	4	1	147	0	2
	$Motif4$	1	3	0	178	7
	$Motif5$	0	1	0	1	170
	other	270	11	42	5	16
separate limma	$Motif1$	53340	21	12	22	5
	$Motif2$	0	16	0	0	4
	$Motif3$	0	0	14	0	2
	$Motif4$	0	0	0	17	1
	$Motif5$	0	0	0	0	0
	other	535	163	174	161	188
all concord	$Motif1$	43	36	49	4	
	$Motif2$	0	0	0	0	0
	$Motif3$	0	0	0	0	0
	$Motif4$	0	0	0	0	0
	$Motif5$	17	157	164	151	196
	other	0	0	0	0	0
full motif	$Motif1$	53578	15	11	13	1
	$Motif2$	0	156	0	0	2
	$Motif3$	3	0	146	0	1
	$Motif4$	1	2	0	166	4
	$Motif5$	0	0	0	0	136
	other	293	27	43	21	56
eb1	$Motif1$	47986	24	14	18	0
	$Motif2$	3	47	0	0	5
	$Motif3$	23	1	42	0	1
	$Motif4$	10	0	0	69	1
	$Motif5$	3	0	0	0	38
	other	5850	128	144	113	155
SAM	$Motif1$	53851	120	138	116	89
	$Motif2$	0	0	0	0	0
	$Motif3$	0	0	0	0	0
	$Motif4$	0	0	0	0	0
	$Motif5$	0	0	0	0	0
	other	24	80	62	84	111

Table A.7. Confusion matrix for simulation 7. The column labels indicate the true underlying patterns and the row labels represent the learned configurations.

Method	Differential configuration	$Motif1$	$Motif2$	$Motif3$	$Motif4$	$Motif5$
CorMotif	$Motif1$	52442	3	5	4	1
	$Motif2$	6	188	0	0	1
	$Motif3$	10	0	156	0	0
	$Motif4$	5	0	0	187	10
	$Motif5$	0	0	0	0	165
	other	1412	9	39	9	23
separate limma	$Motif1$	51999	7	24	5	4
	$Motif2$	0	0	0	0	0
	$Motif3$	0	0	0	0	0
	$Motif4$	0	0	0	0	0
	$Motif5$	0	0	0	0	0
	other	1876	193	176	195	196
all concord	$Motif1$	53859	27	49	18	3
	$Motif2$	0	0	0	0	0
	$Motif3$	0	0	0	0	0
	$Motif4$	0	0	0	0	0
	$Motif5$	16	173	151	182	197
	other	0	0	0	0	0
SAM	$Motif1$	53812	108	145	110	100
	$Motif2$	0	0	0	0	0
	$Motif3$	0	0	0	0	0
	$Motif4$	0	0	0	0	0
	$Motif5$	0	0	0	0	0
	other	63	92	55	90	100

Table A.8. Ranks of known SHH target genes by each method in the SHH analysis.

Gene name	Analysis Method	Study 1	Study 2	Study 3	Study 4	Study 5	Study 6	Study 7
Gli1	*separate limma*	6	7	16	9	7	1369	515
	CorMotif	5	6	7	7	6	930	324
	all concord	9	9	9	9	9	9	9
	full motif	5	7	7	4	5	809	308
	SAM	7	6	17	9	10	1627	583
	eb1	33396	25	36	24	24	1828	720
Ptch1	*separate limma*	7	19	4	4	2	783	19
	CorMotif	6	20	8	4	3	495	12
	all concord	5	5	5	5	5	5	5
	full motif	7	16	4	3	2	409	14
	SAM	6	18	5	4	2	964	25
	eb1	13455	8	6	9	4	1464	289
Ptch2	*separate limma*	273	607	9996	1527	458	2530	117
	CorMotif	140	437	462	356	264	1848	69
	all concord	40	40	40	40	40	40	40
	full motif	145	450	482	285	256	1686	70
	SAM	303	630	9066	1431	468	2488	95
	eb1	7331	579	838	727	433	418	161
Hhip	*separate limma*	105	25	31	580	2964	13452	6
	CorMotif	61	19	27	264	652	9259	2
	all concord	22	22	22	22	22	22	22
	full motif	58	22	28	249	632	8529	2
	SAM	107	24	20	597	2903	16223	7
	eb1	6111	32	10	353	326	7462	131
Rab34	*separate limma*	927	553	299	577	396	15782	241
	CorMotif	324	401	164	176	261	10418	150
	all concord	160	160	160	160	160	160	160
	full motif	386	372	139	194	274	9546	151
	SAM	953	613	450	619	430	15923	171
	eb1	1371	1333	1042	1130	1074	12564	1019
Hand2	*separate limma*	34351	11862	6647	6061	196	20672	44939
	CorMotif	3601	3394	2794	1036	544	13371	17909
	all concord	4987	4987	4987	4987	4987	4987	4987
	full motif	3327	3021	2460	917	550	12585	14457
	SAM	34455	12375	8381	6582	207	22592	44945
	eb1	28270	2191	3040	1650	571	23269	33457
Hoxd13	*separate limma*	6805	7572	1893	10644	12	26047	9676
	CorMotif	1990	2371	1746	1223	93	15204	5734
	all concord	933	933	933	933	933	933	933
	full motif	1943	2490	1246	1064	88	14041	4722
	SAM	6724	7763	2684	10553	12	27578	8579
	eb1	6919	804	696	641	14	26742	12464

Table A.9. Comparison of computation time. The time is shown in the unit of seconds. In some cases, the time is also converted to hours (hr) and the converted time is shown in parentheses. For *CorMotif* and *eb10best*, the displayed number includes the time used to search for the optimal motif number K. For these two algorithms, the average computation time per K (i.e., the mean time required for a single K) is also shown as "CorMotif (mean)" and "eb10best (mean)". "limma (original)" corresponds to the original limma without using the EM algorithm to declare differential expression.

Analysis Method	Simulation 5	Simulation 6	Simulation 7	SHH
CorMotif	2038.81 (0.57hr)	5037.39 (1.40hr)	5552.32 (1.54hr)	8760.06 (2.43hr)
CorMotif (mean)	339.80	719.63	694.04	1251.44
all concord	3.27	3.27	3.52	7.58
separate limma	28.33	44.36	532.70	1025.77
limma (original)	6.37	7.65	20.65	21.57
full motif	80.87	508.47	fail to run	1844.07
SAM	9.27	23.24	48.86	20.48
eb1	196.29	25034.95 (6.95hr)	fail to run	311.49
eb10best	53329.04 (14.81hr)	fail to run	fail to run	fail to run
eb10best (mean)	5332.90	fail to run	fail to run	fail to run
